# High phenotypic diversity correlated with genomic variation across the European *Batrachochytrium salamandrivorans* epizootic

**DOI:** 10.1371/journal.ppat.1012579

**Published:** 2024-10-16

**Authors:** Moira Kelly, Christina A. Cuomo, Wouter Beukema, Salvador Carranza, Jesse Erens, Marleen Foubert, Zhimin Li, Stefan Lötters, Vanessa Schulz, Sebastian Steinfartz, Sarah Van Praet, Michael Veith, Frank Pasmans, An Martel

**Affiliations:** 1 Wildlife Health Ghent, Faculty of Veterinary Medicine, Ghent University, Salisburylaan, Merelbeke, Belgium; 2 Infectious Disease and Microbiome Program, Broad Institute of MIT and Harvard, Cambridge, Massachusetts, United States of America; 3 Reptile, Amphibian and Fish Conservation Netherlands (RAVON), ED Nijmegen, the Netherlands; 4 Institute of Evolutionary Biology (CSIC-UPF), Barcelona, Spain; 5 Department of Biogeography, Trier University, Trier, Germany; 6 Technische Universität Braunschweig, Zoological Institute, Braunschweig, Germany; 7 University of Leipzig, Institute of Biology, Molecular Evolution and Systematics of Animals, Leipzig, Germany; University of Michigan Health System, UNITED STATES OF AMERICA

## Abstract

Recognizing the influence of pathogen diversity on infection dynamics is crucial for mitigating emerging infectious diseases. Characterising such diversity is often complex, for instance when multiple pathogen variants exist that interact differently with the environment and host. Here, we explore genotypic and phenotypic variation of *Batrachochytrium salamandrivorans* (*Bsal*), an emerging fungal pathogen that is driving declines among an increasing number of European amphibian species. For thirteen isolates, spanning most of the known temporal and geographical *Bsal* range in Europe, we mapped phenotypic diversity through numerous measurements that describe varying reproductive rates *in vitro* across a range of temperatures. *Bsal* isolates are revealed to have different thermal optima and tolerances, with phenotypic variation correlating with genomic diversity. Using a mechanistic niche model of the fire salamander (*Salamandra salamandra*) as an example, we illustrate how host steady-state body temperature and *Bsal* thermal range variation may influence pathogen growth through space and time across Europe. Our combined findings show how the identity of emergent pathogen variants may strongly influence when and which host populations are most at risk.

## Author summary

In 2013, a new, salamander-killing fungus, *Batrachochytrium salamandrivorans* (*Bsal*) was discovered in the Netherlands. Since then, *Bsal* outbreaks have occurred in a growing number of countries and amphibian species across Europe, causing rapid population collapses. A lot remains unknown about this pathogen, with most studies so far focusing on the type strain from the first outbreak site. Here we show that strains from other outbreaks behave differently and may threaten different amphibian populations at different times. We found that observed phenotypic variation correlated with genotypic variation and identified molecular functions and biological processes under selection across the European *Bsal* epidemic. As both the fungus and the amphibian hosts are very temperature sensitive, we tested *Bsal* growth across a range of temperatures. We found strains displayed different thermal ranges and optimal temperatures and combined these thermal growth curves with models of amphibian host body temperatures to predict strain geographical and temporal ranges. Our findings help to explain observed *Bsal* epidemiology and provide insights into how strain identity strongly influences when and which host populations are most at risk, with important implications for *Bsal* research and amphibian conservation efforts.

## Introduction

Emerging microbial pathogens often evolve at rates far exceeding their host [1]. Small alterations in pathogen dynamics can result in dramatic epidemiological shifts when entire populations are susceptible to a novel disease. Host-pathogen dynamics are especially complicated for emerging multi-host infections in wildlife, which may affect various host species across diverse ecological systems. As such, knowledge about how wildlife disease dynamics are shaped by interactions between hosts, host microbiome, their environment [2], and pathogen variation remains limited.

The chytridiomycosis pandemic in amphibians exemplifies such a dynamic system. Caused by two emergent fungal pathogens, *Batrachochytrium dendrobatidis (Bd)* and *B*. *salamandrivorans (Bsal)*, chytridiomycosis has contributed to declines in more than 500 species [3] in diverse environments across six continents [4]. *Bd* comprises multiple phylogenetic clades [4], with distinct lineages showing different proteomic and phenotypic profiles [5,6], associated with varying patterns of virulence and host preference. Even recently diverged lineages may range in virulence from rapidly fatal to partially protective [6,7]. In addition to pathogen variation, disease dynamics can be dramatically influenced by host susceptibility, with amphibian hosts ranging from rapidly succumbing to fatal chytridiomycosis [8], tolerance [9,10] or resistance [11] to infection. Host-pathogen interactions are also extensively influenced by biotic and abiotic variation (including pollution [12], host microbiome [2], seasonality [13], and temperature [14–17]).

First identified in 2013 [18], *Bsal* epidemiology is currently less well described. However, it also infects a broad range of amphibian species, causing most disease in urodele species [18,19]; for instance, *Bsal* introduction in a fire salamander (*Salamandra salamandra*) population has been linked to a 90% population reduction within 6 months [20]. Relative to *Bd*, the *Bsal* epidemic currently appears more geographically restricted [21]. However, a recent study of nine *Bsal* isolates from the European outbreak found surprising levels of genetic diversity, and isolate-specific metabolic capacities [22]. Given that phenotypic studies to date have been limited to the type strain (the isolate upon which *Bsal* was first described, BundBos2013), nothing is known of how genetic variation may translate into divergent, epidemiologically relevant phenotypes. In other words, current risk assessments and mitigation measures consider only the type isolate, which may not accurately reflect the threat of all isolates collectively.

Therefore, the goal of this study was to map phenotypic variation in *Bsal* isolates spanning the known temporal and geographical range of the European *Bsal* epidemic (n = 13, Table A and Fig A in S1 Text). Given that both likelihood of infection and disease development is dose-dependent [20,23], we particularly focus on characteristics influencing zoospore density, along with known traits associated with virulence in *Bd*, such as sporangia size. We analyse whether phenotypic and genotypic variation is correlated, along with the phylogenetic relationships of newly described *Bsal* isolates and how these insights affect our knowledge of *Batrachochytrium* species. In order to capture some of the variability seen in natural pathogen-host systems, we characterize the differential responses of isolates to one of the most influential abiotic factors–temperature [24,25]. Converting growth curves at variable temperatures to differential thermal performance curves, we implement the latter in a mechanistic niche model that describes fire salamander steady-state body temperature to illustrate how *Bsal* thermal variation may influence pathogen growth through space and time across Europe. Our combined findings show how the identity of emergent pathogen variants may strongly influence when and which host populations are most at risk. There are important insights, for both scientists working with these isolates (variable lifecycle lengths and optimal conditions), and for planning effective monitoring and mitigation efforts.

## Methods

### Ethics statement

Infected animals were collected from outbreaks sites in Belgium under Permits from the Service Public de Wallonie Agriculture, Ressources naturelles et Environnement to transport wild animals for diagnosis: 2016/RS/n°34 avenant2 and 2020/RS/n°01. Infected specimens from the Eifel2019 locality were collected under permit 425-104-778-0003/2020 issued by the Struktur- und Genehmigungsdirektion Nord, Koblenz, Germany. Permits were granted by Untere Naturschutzbehörde Essen (Az 59-5-1-53 (1080)), Mülheim an der Ruhr, Bochum, Ennepe-Ruhr-Kreis, Dortmund, Oberhausen; we are also grateful to the Landesamt für Natur, Umwelt und Verbraucherschutz Nordrhein-Westfalen for ethics clearance of swabbing procedures (Az 1–02.05.40.18.039 from 13 April 2018).

### Phenotypic experiments

The type strain of *Bsal* (BundBos2013) observes a life cycle of two main life-stages (motile zoospores and zoosporangia) that is completed in 5 days at 15°C [26]. We collected *in vitro* phenotypic data for 13 *Bsal* isolates (Table A in S1 Text), including zoospore count, sporangia count, sporangia size (mean, minimum, maximum and standard deviation) and fecundity at seven different temperatures (0°C, 4°C, 10°C, 15°C, 20°C, 25°C, 30°C, as in Beukema et al. (2020) [24]).

Spores were collected from the isolates in culture in TGhL medium. Sixteen hours before experimental set up culture medium was replaced with 0.8% Tryptone TGhL medium and 50ml of TGhL medium was placed at the test temperatures (0°C, 4°C, 10°C, 15°C, 20°C, 25°C, 30°C) to equilibrate. Zoospores were collected in 0.8% tryptone TGhL medium, filtered using a 10μm cell strainer (Pluristrainer, https://www.pluriselect.com) and diluted in to the desired concentration of 5x10^5^ zoospores per ml. One millilitre of this zoospore solution was then added to 2 wells of three separate 24 well plates per temperature, totalling 6 wells at each temperature per isolate. These plates were then wrapped in aluminium foil to standardize daylight exposure and placed at one of the test temperatures for 3 hours. After three hours the medium was removed from the wells and replaced with 1ml 1.6% tryptone TGhL acclimatised to experimental temperature in all 3 plates, this was designated as time zero, as all non-attached zoospores had been removed–attached zoospore counts at this time point were used to quantify initial zoospore attachment. One plate was then removed and processed for qPCR [27], the two remaining plates were photographed using a Leica DMi1 microscope with camera, taking photographs of 3 random positions of the base of the well. The plates were then wrapped in aluminium foil again and returned to the experimental temperature. Photographing was repeated on day 3, 5, 7 and 10. On day 3, three random positions of the surface of the well medium were also photographed, to identify non-motile spores [20]. On day 5, 1ml of 16% tryptone TGhL media acclimatised to the experimental temperature was added to each well, at this point the depth of media precluded photographing the surface of the well. Plates were transported to photographing in insulated boxes, on ice for the case of 0 and 4°C plates, and replaced immediately after photographing. Photographing each plate was performed as quickly as possible, with each plate only removed from the experimental temperature experiment for less than 15 minutes. The experiment finished on day 10 with all wells being processed for qPCR, which estimates the number of *Bsal* cells present within a solution based on the number of copies of the 5.8S rRNA gene in comparison to solutions with known quantities of *Bsal* zoospores.

A 1000x1000 pixel square centred at pixel 500x500 was analysed from each photo using the software ImageJ [28]. The number of zoospores (zoospore count), the number of sporangia (sporangia count) and the area of each sporangia was measured (summed to identify sporangia coverage per image); and the minimum, maximum, mean and standard deviation of sporangia size was calculated for each photograph. All such analyses was performed blinded. The counts were performed by two “observers”, with 93% of photographs analyzed by observer 1 and 7% of photographs analysed by observer 2, images were randomly assigned to observers. To negate the influence of subjectivity in designating a fungal body as “spore” or “sporangia”, observer was included in mixed effect models as a random effect. We also calculated fecundity (a measure associated with virulence in *Bd* isolates [5], calculated as the number of zoospores/sporangia coverage and here standardized to account for initial zoospore attachment by dividing zoospore counts by the average zoospore count of that well at t = 0).

The large number of technical repeats at a variety of temperatures and high zoospore concentration necessitated a large volume of zoospore solution- requiring all isolates to sporulate sufficiently at the same time. As our results show, these isolates have different length lifecycles, and so it proved impossible to get all isolates to sporulate at the same time when subculturing on the same day. We repeated the experiment a total of four times, with the BundBos2013 isolate present in all experiments, facilitating the inclusion of experiment as a random effect in our mixed effect models. All other isolates were included in two experiments, except Captive2015-2, Eifel2019 and Rob2014 for which sufficient zoospores were only collected in one experiment.

All statistical analysis of phenotypic data were performed in R studio with R version 4.0.0. Growth curves were fitted to total (spore and sporangia) counts per well using the GrowthCurver [29] package version 0.3.1 and the intrinsic growth rate, r, generation time, *t_gen*, and carrying capacity, k, of each well extracted. We also fit growth curves to qPCR results, but the accuracy of these curves was limited as this data was only available for day 0 and day 10, and the growth curves based on observational counts illustrated that the cultures at 10°C and 15°C had reached carrying capacity much earlier in the experiment whereas lower temperatures were still in exponential growth phase. A range of polynomial curves (1 to 4 degrees) were fit to the extracted *r* values using lm() and poly() functions in R, the best fitting curve was identified using anova and the coefficients extracted (see Table B in S1 Text).

We performed a principle component analyses (PCA) on the zoospore count, sporangia count and mean sporangia size across all times using prcomp() in R. As this method requires a complete numeric matrix (with no missing values), for each of these three metrics (spore count, sporangia count and mean sporangia size), we included 4 data points per temperature and time point–equivalent to the 4 wells at each time point, for isolates that were in multiple experiments, the mean value for each of these datapoints was used. We performed a K-means clustering analysis on the phenotypic PCA output, initially utilising fviz_nbclust() to calculate the silhouette and gap statistics, which identified 2 clusters as optimal. We then used the function eclust(), trying both the Euclidean and Manhatten distances, with 2 clusters to identify isolate clusters. We found both distance metrics identified the same clustering with the same mean silhouette statistic.

All data and analysis scripts are available on the DRYAD database https://doi.org/10.5061/dryad.4xgxd25j5 [30].

### Geographical predictions of *Bsal* growth relative to climate and host body temperature

The observed differences in thermal growth between isolates may drive variation in the amount and timing of growth peaks during the year, relative to local climate and host body temperature. To illustrate this variation, we built on the mechanistic model of Beukema et al. (2020)[24], which simulates average fire salamander steady-state body temperature across its distribution, for each day of the year. Beukema et al. (2020) [24] generated predictions of where, during which time of the year, peak growth of the type strain BundBos2013 may occur. Here, we extend these predictions using thermal growth data of all thirteen *Bsal* isolates.

### Summary of Mechanistic Niche Modelling approach

Applying the mechanistic model from Beukema et al. (2021) [24], we first built a microclimate model using the NicheMapR package version 2.0.0 [31], which estimates ground-level conditions available to a given (host) species. Fourteen topographic and climatic variables derived from a Digital Elevation Model (SRTM v4.1) and the ERA5 climate reanalysis dataset (Copernicus Climate Change Service, 2017) were used to inform the model. Climate data spanned the period ranging between 01-01-2010 and 31-12-2018 to match with *Bsal* emergence, and were obtained at hourly interval. The microclimate model was then used as input for the NicheMapR ectotherm module [32] set to iteratively calculate daily average steady-state body temperature throughout the year for an organism of 40g with a lizard-like shape. Evaporative water loss was taken into account by considering the surface of the organism as a free-water exchanger. Nocturnal and crepuscular activity was assumed, and the percentage of shaded conditions was set between 90 and 100%. Finally, we assumed that the organism would retreat up to 20cm underground when relative humidity fell below 85%, after which its body temperature adopted that of the soil. Refer to Beukema et al. (2021)[24] for details on data preparation and model parameterization.

The thermal growth curves of the different *Bsal* isolates were subsequently applied to the fire salamander steady-state body temperature model. We restricted growth to 0–25°C as both our experiment and previous studies [26] found temperatures of 25°C or above incompatible with growth. We then predicted *Bsal* growth across the fire salamander distribution for each day of the year. No relationship between temperature and pathogenicity was assumed [20].

### Genomic data analysis

Following the identification of surprisingly high levels of genetic variation within the European *Bsal* epidemic [22], we explored genetic variation and phylogenetic relationships within the expanded set of new *Bsal* isolates. Genomic DNA was extracted from isolates Captive2015-3, Eifel2019 and Essen2019, and combined with genomic data from 9 other *Bsal* isolates (Table A in S1 Text). DNA was extracted from cultures of comparable passage numbers, all isolates were cryopreserved, revived, and cultured under identical conditions. The yeast protocol for Qiagen 100G genomic tips was used, while dsDNA concentration was checked using the Qubit dsDNA HS Assay Kit. Whole genome sequencing was performed using Illumina Novaseq6000 PE150 technology (Captive2015-3, Eifel2019, Essen2019) with the NEBNext Ultra II kit and no PCR cycles used in library prep; and PacBio Sequel technology (Essen2019), with library prep, quality checks, and sequencing performed by Novogene UK (25 Cambridge Science Park, Milton Road, Cambridgeshire, CB4 0FW, United Kingdom). Data was visually assessed using FastQC [33].

### Genome assembly, gene prediction and variant annotation

A *de novo* assembly for the Essen2019 isolate was assembled, checked and annotated using the same approaches as in Kelly et al. (2021) [22]. This *de novo* assembly was compared to published assemblies for *Bsal* isolates (BundBos2013, Luik2014, Captive2015-1, Rob2015, Captive2015-2, and Catalan2018; Table A in S1 Text).

### Phylogenetic analysis

We inferred phylogenetic relationships on both *Bsal-*only and *Bsal-*plus-*Bd* datasets to allow for a better comparison of only *Bsal* variation as well as divergence on the genus level of *Batrachochytrium*.

For the *Bsal-*only analysis (including in the case of the phylogeny for Fig 4A the BdJel423 isolate) we aligned Illumina reads from all *Bsal* isolates to the primary contigs of the BundBos2013 assembly (GCA_021556675.1) and performed variant calling and filtering using GATK [34] v4.1.8 and bcftools [35]. This identified 106,942 SNPs, which we filtered with SNPRelate [36] v1.22.0 and a Linkage Disequilibrium threshold of 0.2, leaving 4,600 SNPs. A PCA was performed on both this LD-filtered SNP subset and the 106,942 SNP set. We also inferred a phylogeny with RAxML-NG [37] v. 1.0.3 (Fig 4A). Here we included *Bd*Jel423 (Bioproject PRJNA13653) as an outgroup, we aligned all reads to the BundBos2013 assembly (GCA_021556675.1) and called and filtered variants as described using GATK [34] v4.1.8 and bcftools [35], leaving 108,776 SNPs. We inferred ML trees, bootstrap trees, checked for convergence and calculated bootstrap support following best practises [38]. We further inferred a single copy core ortholog tree (based on the trees of 2,798 orthogroups) using OrthoFinder [39] on the genome assemblies of all published *Bsal* isolates, plus our assembly of the Essen2019 isolate, and including the *Bd* Jel423 isolate annotated assembly (Bioproject PRJNA13653, Accession number GCA_000149865.1) as an outgroup. For aneuploidy and coverage analyses we aligned Illumina reads to the BundBos2013 assembly (Accession number GCA_002006685.2) and performed variant calling and filtering as described above. To describe structural variants (>50bp), we aligned isolate assemblies to the BundBos2013 assembly (Accession number GCA_002006685.2) using nucmer from mummer with the parameters “-maxmatch -l 100 -c 500”, according to the Assemblytics manual, and then ran an assemblytics analysis with default input parameters.

For *Bsal-*plus-*Bd* analyses, we aligned all *Bsal* isolates, along with a select subset of *Bd* isolates (Table B in S1 Text) to the BundBos2013 and *Bd* Jel423 assemblies for variant calling. We aimed to select a subset of *Bd* isolates that balanced with the *Bsal* isolates. We therefore randomly identified 9 *Bd* isolates with Illumina data present on NCBI, that were all isolated in Europe and representing the three clades identified within Europe (*Bd* GPL, CAPE and CH lineages). All reads were then aligned to the *Bsal* BundBos2013 assembly primary contigs, and also the published *Bd* Jel423 assembly (Bioproject PRJNA13653, Accession number GCA_000149865.1). Variant calling and SNP filtering were performed as described above, identifying 478,996 SNPs when aligned to the BundBos2013 assembly and 227,230 SNPs when aligned to the *Bd* Jel423 assembly. Tajima’s D calculations were performed on the SNPs called to the BundBos2013 assembly, using PopGenome version 2.7.5 in R version 4.0.0, and dN/dS values were calculated with yn00 from PAML.

## Results

### High between-isolate phenotypic variation during the *Bsal* lifecycle

**Fig 1:**
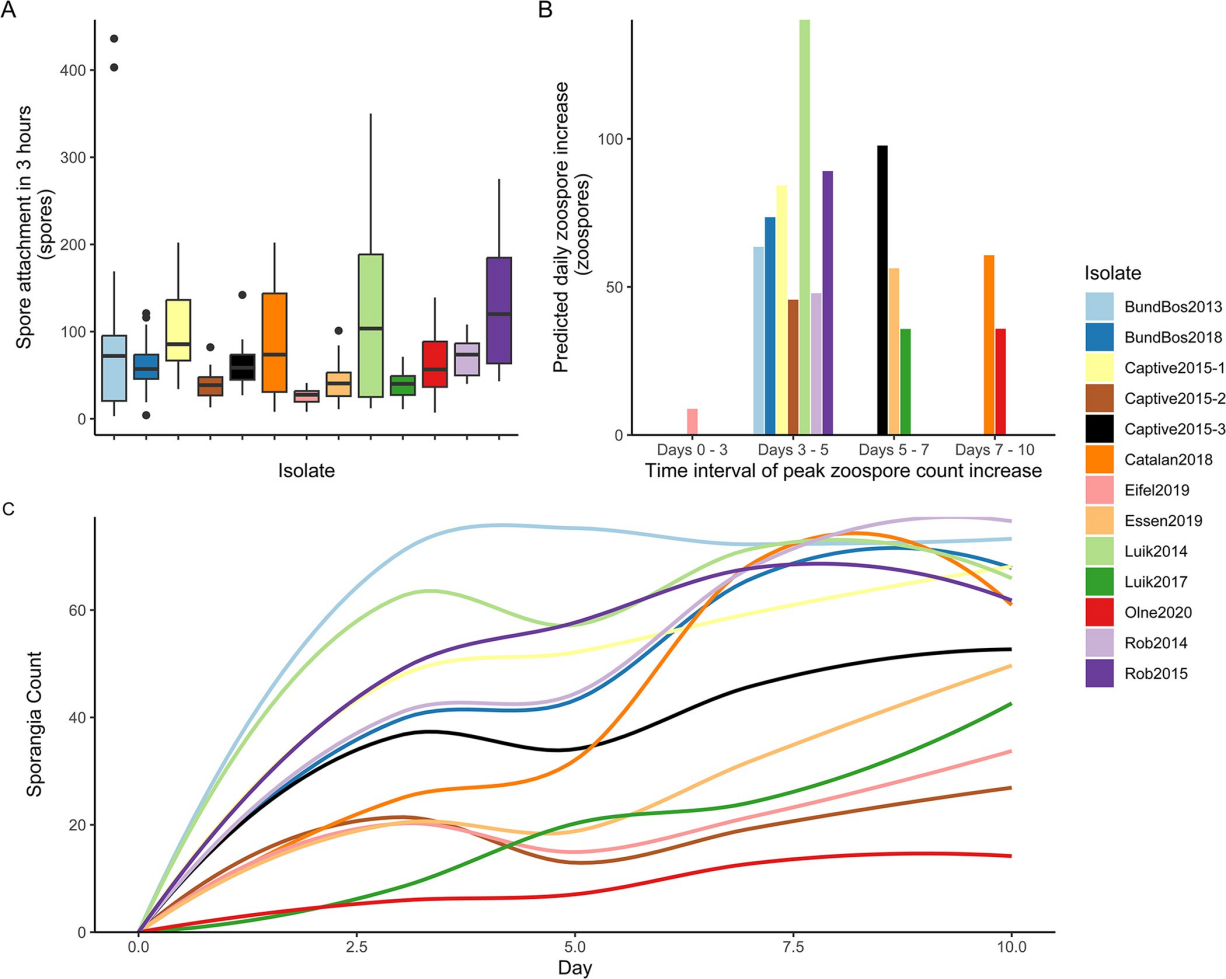
Batrachochytrium salamandrivorans zoospore attachment, zoospore production and zoosporangia maturation at 15°C. A) Boxplot of the number of zoospores attaching within three hours after introduction of a solution of standardised zoospore concentration. B) Predicted time and size of maximum daily increase in zoospore concentration–indicating the predicted timing of sporulation for each isolate, thus providing an indication of generation time. Here the y axis represents the predicted daily increase in the zoospore count. We predict the timing of sporulation (timed from t0 of experiment) using simulated data from a generalise linear model with a negative binomial distribution and the formula zoospore count ~ isolate*day (R^2^ = 0.8), with day treated as a factor due to the observed non-linear relationship of zoospore count. The time period with the steepest daily increase in zoospore count was identified as the predicted period of sporulation. C) Sporangia count over time per isolate.

We observed intraspecific variation in various aspects of the *Bsal* lifecycle (Fig 1), including speed and degree of zoospore attachment, time to sporangia maturation, timing and number of zoospores released, sporangia size, and fecundity.

Attachment of zoospores is essential for encysting and maturation into zoosporangia. While rapid attachment may be associated with rapid colonisation, conversely, slow attachment of the motile zoospore could result in increased dispersal. To assess zoospore attachment we quantified how many zoospores attached within 3 hrs of introduction of inoculate of standard concentration (5x10^5^ zoospores per ml) (see Fig 1A). The Rob2015 and Luik2014 isolates demonstrated higher numbers of zoospore attachment at 15°C, while fewer zoospores of the Captive2015-2, Eifel2019, Essen2019 and Luik2017 isolates attached (general linear model with negative binomial distribution, structure spore count at 15°C ~ isolate, Observations = 282, Marginal R2 = 0.213, see Fig B in S1 Text).

Following attachment, zoospores encyst and mature into sporangia. As such, zoospore counts over time are non-linear, decreasing when zoospores mature to sporangia, and increasing when sporangia sporulate and release more spores (Fig C.A and C.B in S1 Text). We therefore fitted a negative binomial model (spore_count ~ Isolate*Day, treating time as a discrete variable due to the non-linear relationship with time, Observations = 1353, R^2^ = 0.849) and simulated data to visualize the relationship of zoospore count over time for each isolate (Figs C.C and D in S1 Text). Some isolates (e.g. Luik2014), showed a clear time period with a steep increase in zoospore count, suggesting highly synchronised sporulation *in vitro*. Isolates varied in both the timing of sporulation (and therefore lifecycle length), and the magnitude of zoospore increase at sporulation. Most isolates showed lower zoospore counts than BundBos2013 (Fig C.D in S1 Text), with only the Rob2015 and Luik2014 isolates showing higher zoospore counts overall (IRR = 1.60, CI = 1.19–2.18, p = 0.002 and IRR = 1.41, CI = 1.05–1.92, p = 0.026).

Zoosporangia counts at 15°C (Fig E in S1 Text) show considerable intra-isolate variation in doubling times (*t_gen*) and growth rate (*r*) [29]. A generalized linear model (glm) of *t_gen* measurements (structure *t_gen* ~Isolate) illustrated significantly longer generation times in Essen2019 (coef. est. = 1.97, CI = 1.05–2.89, p = <0.001), Eifel2019 (coef. est. = 2.18, CI = 1.14–3.23, p = <0.001), Luik2014 (coef. est. = 1.62, CI = 0.70–2.54, p = 0.001), and Luik2017 (coef. est. = 1.46, CI = 0.42–2.51, p = 0.008) isolates, with nearly all isolates showing slower zoosporangia count growth rates than BundBos2013 (glm with structure *r* ~ Isolate, see Table C in S1 Text). Thus, the BundBos2013 isolates shows some of the highest zoospore counts and fastest rates of maturation to sporangia (Fig 1C). We observed less variation, both within and between isolates, in total counts of sporangia and zoospores. Here, BundBos2013 had a slower *r* (coeff. est. = 2.29, CI = 1.37–3.20, p = <0.001) and longer *t_gen* than Luik2014 (coeff. est. = -2, CI = -3.39 –-0.60, p = 0.007), and indeed a slower t_gen than all isolates except Captive2015-2, Eifel2019, Luik2017 and Olne2020 (Table C and Fig F in S1 Text).

Sporangia size, which is associated with virulence in *Bd* [5], also varied considerably between isolates. The BundBos2013 isolate displayed the fastest increase in zoosporangia coverage, and most isolates displayed lower total zoosporangia coverage over time than the BundBos2013 isolate (mixed effect model fit with lmer, with structure sporangia coverage ~ Isolate * day with experiment as a random effect, Fig G in S1 Text). However, when considering fecundity at 15°C, the BundBos2013 isolate appears representative of the majority of isolates with Catalan2018, Captive2015-2 and Luik2017 displaying higher fecundity (p = 0.022, p = 0.023 and p<0.001 respectively, from a generalised linear model with structure Fecundity ~ Isolate * Day, considering time as a discrete variable, Fig H and Table D in S1 Text).

### Thermal tolerance differs between *Bsal* isolates and variably influences their lifecycle

Repeating the growth tests at seven different temperatures (0°,4°,10°,15°,20°,25°, and 30°C, see Fig I in S1 Text), we found variation in all growth measurements and variable isolate responses to this temperature gradient.

Spore attachment is a largely active process in *Bsal;* thus the addition of a killed culture results in few to no zoospores attaching. Data from the BundBos2013 isolate indicated mortality after 5 days at 25°C [18]. In fact, high temperatures appear to impact BundBos2013 function within only hours, as nearly no zoospores attach when placed at 30°C (Fig J in S1 Text). Conversely, the Luik2014 isolate has the highest zoospore attachment rates at 30°C, suggesting such temperatures are not so rapidly debilitating or lethal in all isolates.

**Fig 2:**
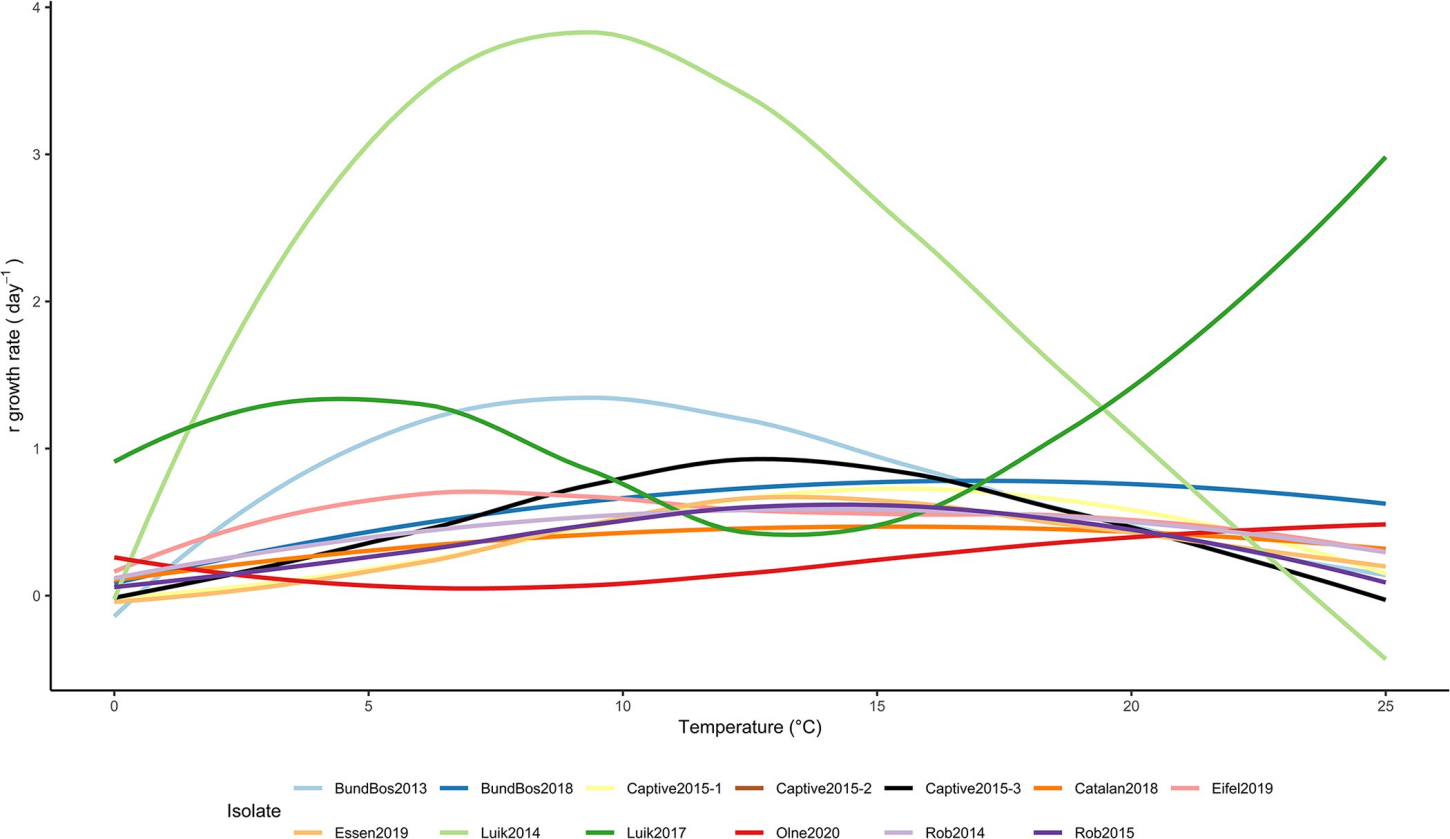
Isolate growth at various temperature regimes. Optimal polynomials fit to growth rates (r value (count.day^-1^), calculated by GrowthCurver considering total (spore + sporangia) counts) vs temperature data (line colour depicts isolate identity). (see Fig K in *S1 Text* for individual plots of growth curves against r values (count.day^-1^) and R^2^ values, and Fig L in *S1 Text* for other growth measures (generation time and carrying capacity) analysed for each isolate).

To characterize how temperature influences growth rates for each isolate, we fitted growth curves to total (zoospore + sporangia) counts [29] and extracted the growth rates, *r*, for each well. No isolate had a linear relationship between growth and temperature, so an optimal polynomial fit for the growth rate measurements with temperature was determined to predict thermal optima and confidence intervals for each isolate (Fig 2, and Table E and Figs K and L in S1 Text). We found highly variable responses to temperature, with estimated optimal growth temperatures ranging from 6°C (Eifel2019 isolate, polynomial R^2^ = 0.35) to 24°C (Luik2017 isolate, polynomial R^2^ = 0.17), and variable estimates of fecundity (Fig M in S1 Text).

Optimal growth curves, calculated from qPCR data (Fig N in S1 Text) also found substantial variation in “optimal” temperatures for growth ranging from 11°C (polynomial R^2^ = 0.60) to 19°C (polynomial R^2^ = 0.90, Figs O and P in S1 Text).

### Varying phenotypic plasticity in response to adverse conditions

**Fig 3:**
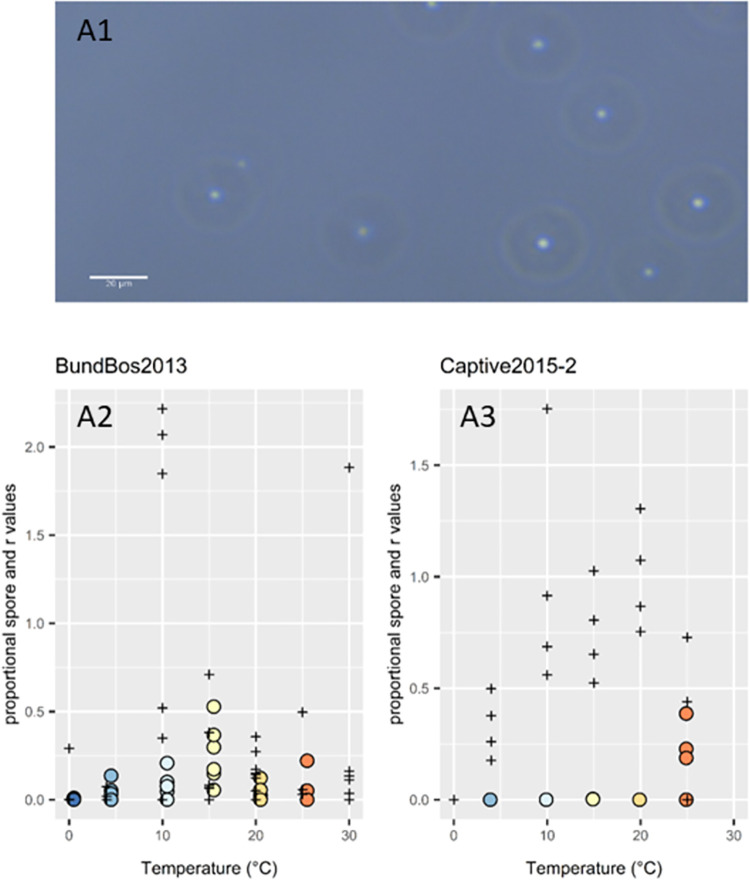
Batrachochytrium salamandrivorans phenotypes: plasticity in zoospore motility. A1) photographic representation, photographed at 20x magnification, scale bars represent 20μm. A2-A3) Scatterplots of standardized growth rates (r = black crosses, calculated from growth curves fit by GrowthCurver, standardized to bring onto a proportional scale as the proportion of immotile spores, by first calculating the average r value for each isolate at each temperature, identifying the maximum average r value for each isolate and dividing the extracted r values by this maximum average r value), with proportion of zoospores that are immotile, floating zoospores (coloured points- colour denotes incubation temperature). These plots are shown for all isolates in Fig R in *S1 Text*.

Previous studies observed the production of a more resistant, non-motile (‘encysted’) zoospore by BundBos2013 [20] (Fig 3A1). We found that for 93% of wells (across all isolates and temperatures), non-motile spores make up less than 20% of total zoospores produced (Fig Q in S1 Text). Yet, we observed large variation even between isolates from the same outbreak site (BundBos2013 and BundBos2018), as BundBos2013 produced the most immotile spores of all isolates while BundBos2018 produced the least (BundBos2013 immotile spore proportion max = 53%, mean = 8.3%, BundBos2018 max = 5.7%, mean = 2.4%). Immotile spores are longer-lived than motile zoospores and are observed *in vivo* [18] as well as in cultures growing under adverse conditions, and were hence hypothesized to represent a resistant phenotypic response, preferentially expressed in conditions adverse to the pathogen [20]. Indeed, five out of six incidences where mean immotile spore proportion (per temperature and isolate) was greater than 20% were far from that isolate’s optimal temperature (four at 20°C and one at 0°C; Fig Q in S1 Text). However, the BundBos2013 isolate produced maximal immotile spores at 15°C, which is also one of the two temperatures at which it grew best (10°C and 15°C). Thus, when comparing the proportion of zoospores that are immotile spores with the exponential growth rate, *r*, a number of isolates (Eifel2019, Olne2020, Luik2017, and Captive2015-2; Fig 3A,) produce a higher proportion of immotile spores at “suboptimal” temperatures where growth rate measurements, *r*, are lower. However, for most isolates the proportion of motile zoospores shows no correlation with *r*, and three isolates (BundBos2018, Captive2015-1, Rob2015) appear to produce more immotile spores at temperatures with higher growth rates (Fig 3A3 and Table F in S1 Text, beta regression model with structure (proportion of immotile spores ~ Isolate * *r* value)). We also observed altered, enlarged sporangia at 20°C, as described in Text A in S1 Text.

### Genotypic variation underlies phenotypic variation

**Fig 4:**
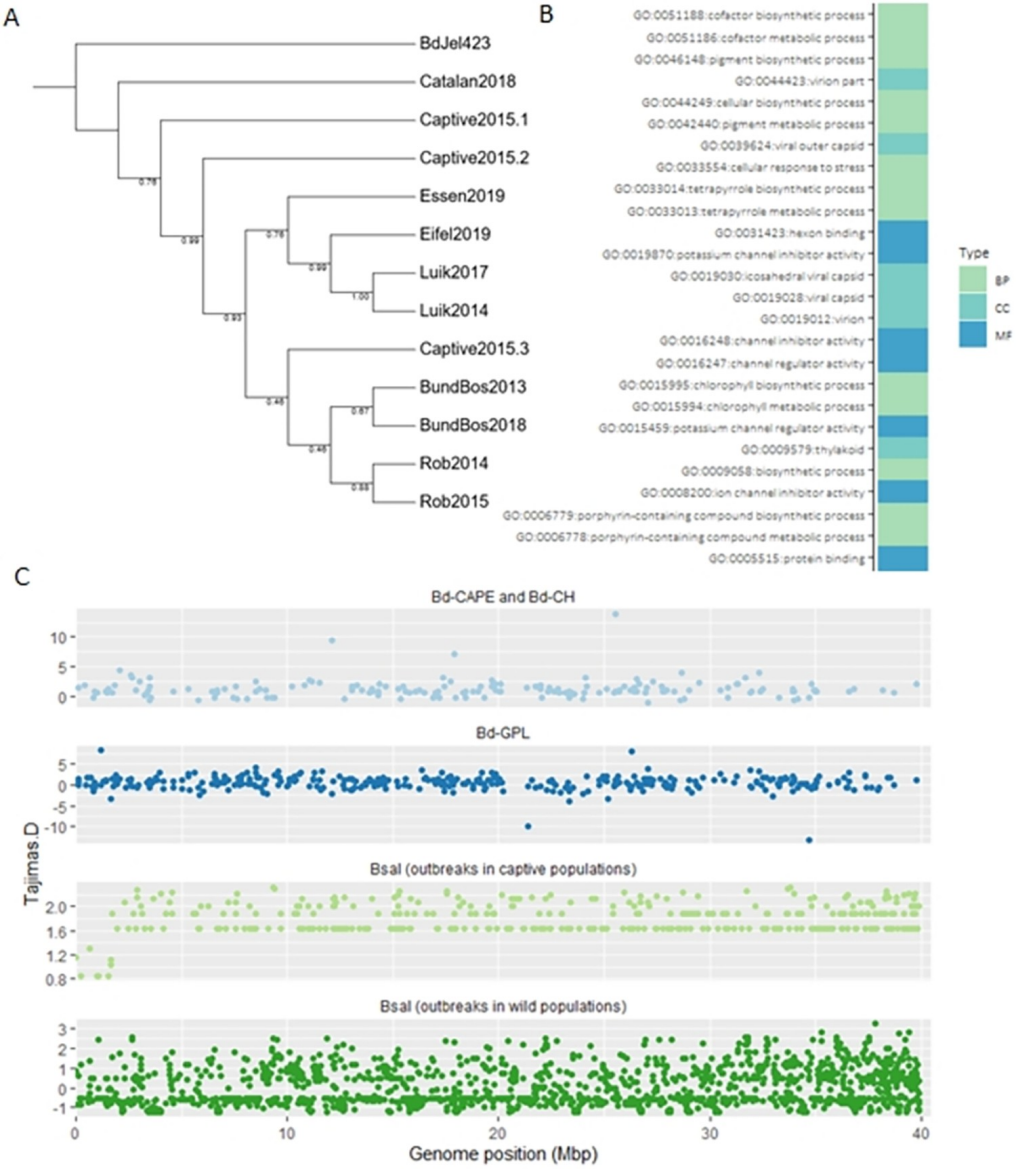
Genomic variation within the European *Batrachochytrium salamandrivorans* epidemic. A) RAxML inferred phylogeny of LD filtered SNPs of all isolates aligned to the BundBos2013 assembly (GCA_021556675.1), including BdJel423 reads as an outgroup, node values denote bootstrap support. B) GO Terms and descriptions enriched in the ortholog clusters within highest 10% of dn/ds values. BP = Biological Process, CC = Cellular component, MF = molecular function. C) Tajima’s D calculations of non-overlapping 10kb windows across BundBos2013 assembly (see Supplementary Methods for details).

There is considerable genomic variation within the European *Bsal* epidemic [22]. Combining whole genome Illumina sequencing of three new isolates (Essen2019, Eifel2019, and Captive2015-3), with published data from 9 other isolates, we first aimed to position these new isolates within the *Bsal* phylogeny. Our RAxML inferred phylogeny was largely consistent with that found in Kelly et al. (2021) [22], and positioned the Eifel2019 and Essen2019 isolates with the Luik2014 and Luik2017 isolates, while the Captive2015-3 isolate clustered with the Rob2014, Rob2015, BunBos2013 and BundBos2018 isolates (Fig 4A).

To better place the variation observed in the European *Bsal* outbreak within the scope of *Batrachochytrium* variation, we also performed variant calling on a combined *Bsal-*and-*Bd* dataset including nine *Bd* isolates (Table B in S1 Text and Methods). Principal Component Analysis (PCA) of *Bsal* and *Bd* isolate SNP variation also indicate the Rob and BundBos isolates clustering together, and the Eifel2019 and Essen2019 isolates clustering with the Luik isolates, along with Captive2015-1, Captive2015-2, and Catalan2018. As low coverage of *Bd* isolates resulted in poor segregation when aligned to the *Bsal* BundBos2013 assembly, we also aligned sequence data for all *Bsal* and *Bd* isolates to the *Bd* Jel423 assembly (Assembly GCA_000149865.1) and compared the PCA, Identity by Descent, and SNPRelate outputs to assess how assembly selection affected the inferred phylogenetic relationships and observed very similar isolate clustering (Fig S in S1 Text). Aneuploidy and repetitive region expansions and contractions have been identified as common sources of genetic diversity in *Batrachochytrium* species [22,40–42]. We therefore estimated ploidy of heterogenous SNPs (Fig T in S1 Text), and repeat region variation (Table H in S1 Text) of 5 *Bsal* isolates aligned to the reference BundBos2013 assembly (GCA_002006685.2). While we still observe a majority of bi- or tetra-ploidy sites, we observe higher rates of trisomy than in the first assessment of ploidy, which considered only the BundBos2013 isolate [41], and do not observe the same pattern of enrichment of trisomy in smaller contigs. Structural variants (50bp or greater), were found to represent between 0.47 and 0.77% of isolate assembly alignments, of which 67–83% were repeat or tandem expansions or contractions.

To identify and compare signatures of demographic history of *Bsal* and *Bd*, we calculated Tajima’s D for non-overlapping 10kbp windows across the BundBos2013 assembly (Fig 4C). We grouped *Bd* isolates according to established phylogenetic clades [4] (as confirmed in our phylogeny) and grouped *Bsal* isolates by captive or wild amphibian population outbreak origin (supported by phylogenetic and phenotypic similarities). As previously observed [4], we found the *Bd* lineages had fluctuating negative and positive Tajima’s D values, of maximum amplitude in the Global Pandemic Lineage (GPL), indicating historical population size fluctuations with signatures of recent selective sweeps or population expansions following a bottleneck (negative Tajima’s D values) along with evidence of balancing selection or sudden population contractions (positive Tajima’s D values). The *Bsal* isolates collected from wild outbreaks also displayed a profile of positive and negative values, consistent with historical demographic fluctuations. By contrast, *Bsal* isolates from captive populations show a much narrower profile, with lower values in early genomic regions, and only positive Tajima’s D values, illustrating perhaps a much more recent population contraction, or consistent balancing selection.

To identify gene orthologs under selective pressure, we calculated dN/dS (ω) for *Bsal* genes identified as ortholog clusters by OrthoMCL and found 2,870 clusters of genes with a signature for positive selection (ω >1). Fisher enrichment tests of Pfam annotations indicated enrichment for a wide range of biological processes and molecular functions (S1 Data), including actin cytoskeleton modification, peptidoglycan modification, cell stress responses and potassium ion channel inhibition; with clusters containing candidate effector proteins such as M36 fungalysin metalloproteases and CAZyme candidates for chitin modifiers CE4 and CBM50 and seven families of CAZymes associated with plant carbohydrate metabolism [22,43]. The clusters within the highest 10% of ω values showed enrichment for potassium ion channel inhibition, cell stress responses and chlorophyll metabolism (Fig 4B). Actin cytoskeleton modification may be important in mediating host cell entry for fungal pathogens [44] while potassium ion channel inhibition can mediate host immune function inhibition by fungal pathogens [45]. Finally, as previous studies had shown significant variation in gene arsenals between isolates, we generated a *de novo* assembly of one isolate (Essen2019), and we found this isolate to be intermediate in the genomic variation observed in the previously published *Bsal* assemblies (Fig U.B in S1 Text).

### Phenotypic variation correlates with genotype

**Fig 5:**
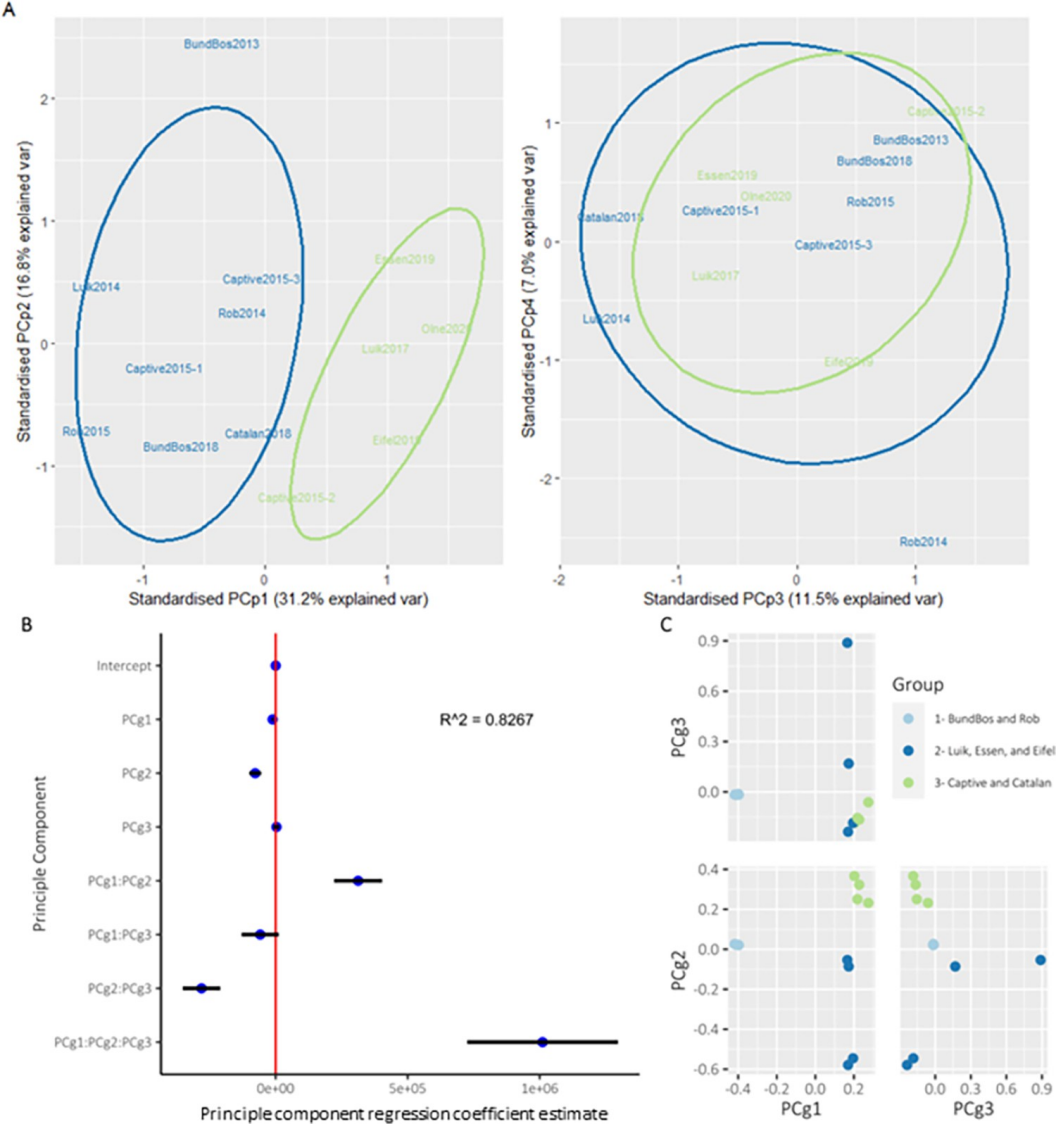
Correlation between genomic and phenotypic variation. A) Principal component analyses (PCA), performed using prcomp() in R, with first four principle components (PCp) explaining 66.5% of variation of phenotypic data (spore counts, sporangia counts and mean sporangia size) across all temperatures and time points. Colour of isolate label and ellipses depict cluster identity according to K-mean clustering analysis. B) Estimates with 95% confidence intervals of coefficients from linear model (model structure PCp1*PCp2*PCp3* PCp4 ~ PCg1*PCg2*PCg3, R^2^ = 0.827) where PCg = genomic principle component and PCp = phenotypic principal component. C) Genomic PCA output. Pairwise scatterplots of first three principal components with isolates colour indicating isolate group as shown in legend positioned in D.

We performed a PCA on phenotypic traits, such as the zoospore count, zoosporangia count and mean sporangia size across all conditions. The first four phenotypic principle components (PCp1: PCp4) captured ~67% of the variation (Fig 5A). Considering variable loadings, we saw that sporangia size loaded heavily positive on PC2, and to a lesser extent PC1, with spore and sporangia counts loading most heavily negatively on PC1. Growth at temperatures of 10°C and below loaded positively on PC3 (Fig V in S1 Text). We performed K-means clustering on the phenotypic PCs, which identified 2 clusters, with isolates from the same genetic clades broadly clustering together (Figs 4A and 5A). Accordingly, we fit a linear model to assess correlation between genomic variation (SNP PCA principle components PCg1-PCg3; see Fig 5C) and the observed phenotypic variation (PCp1: PCp4). We found significant correlation between PCg2 (p = 0.026), PCg1:PCg2 (p = 0.026), PCg2:PCg3 (p = 0.017) and PCg1:PCg2:PCg3 (p = 0.023) and the four PCs of the phenotypic PCA (PCp1-PCp4, Fig 5B). Thus, genetic relatedness appears to correlate with *in vitro* phenotype.

### Isolate variation shapes spatial predictions of *Bsal* growth

**Fig 6:**
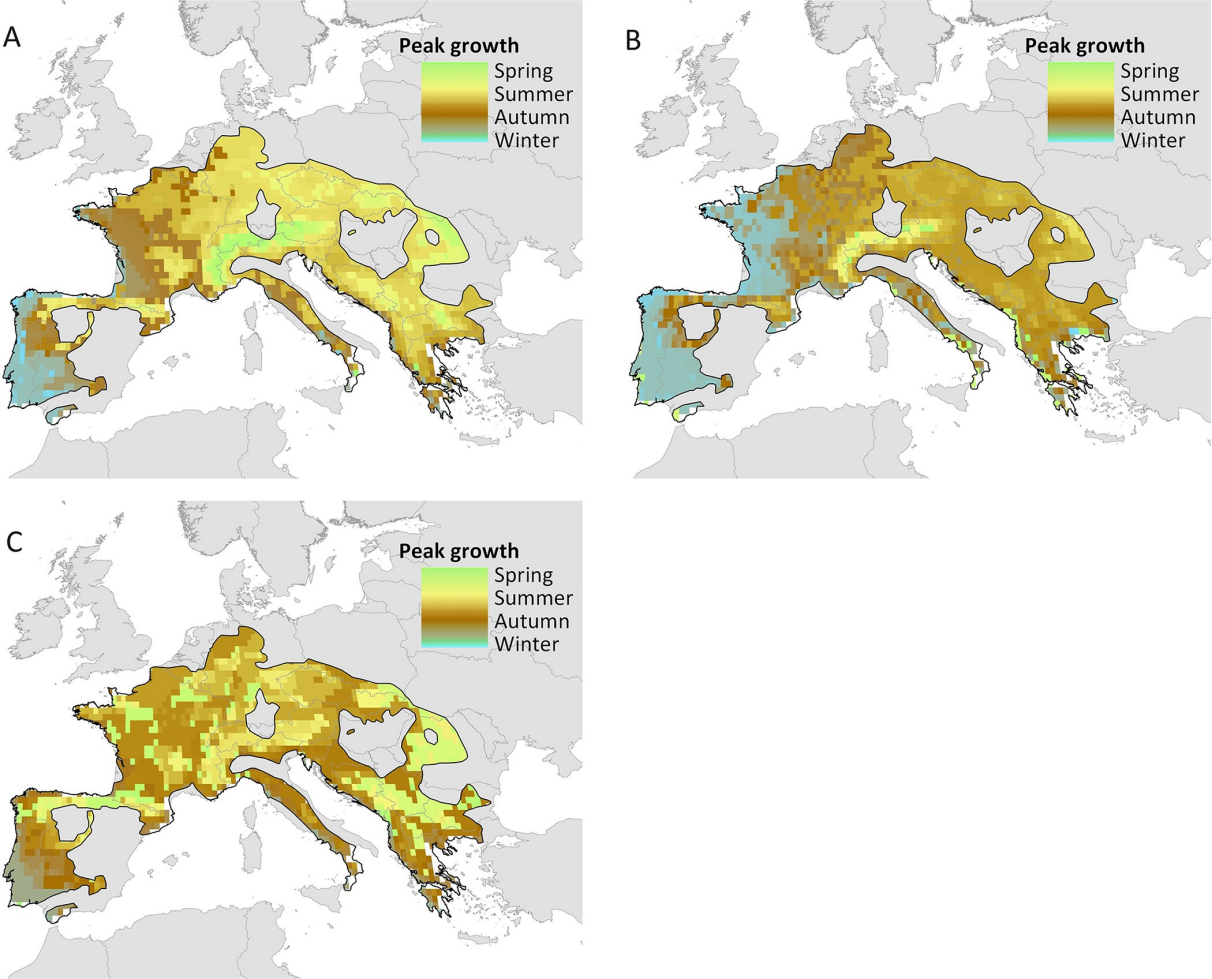
Predicted timings of optimal growth of Bsal across fire salamander (Salamandra salamandra) distribution, coloured per meteorological season for overall interpretability. A) Eifel2019, B) Luik2017, C) Rob2014 pixel colour depicts timing of peak Bsal growth where peak growth occurs in green = spring, yellow = summer, brown = autumn and blue = winter. Generated using shape file from https://www.geoboundaries.org/index.html#getdata.[60].

We generated spatial predictions of per-isolate *Bsal* growth relative to microclimate and fire salamander body temperature following the methods of Beukema et al. (2021) [24]. Our predictions illustrate how differences in thermal growth between isolates may influence the timing and occurrence of growth peaks throughout the year. For instance, the thermal curve for Luik2017 show a preference for growth at both relatively lower (3–6°C) and higher (>20°C) temperatures. Fire salamander body temperatures however rarely rise above 20°C, due to which peak growth of Luik2017 is predicted to occur during relatively lower winter temperatures across Western Europe (Fig 6). The isolate Eifel2019 follows a similar pattern. Conversely, peak growth of other isolates is often predicted to occur in autumn, or, especially in mountain ranges, in spring or summer. As salamander physiology, including behaviour and immune function varies dramatically between seasons, which isolate is introduced to a specific population is therefore likely to have a drastic effect on disease expression.

## Discussion

Understanding wildlife host-pathogen interactions within their environmental context is crucial to predicting which host communities will be most vulnerable. Here, we analyse the genotypic and phenotypic variation among isolates of the emerging amphibian fungal pathogen *Batrachochytrium salamandrivorans (Bsal)*. We found signatures supporting different demographic histories between isolates from outbreaks in captive and wild amphibian populations. Examining gene orthologs for signatures of positive selection highlighted molecular pathways that may be important during *Bsal* infection. Many of the MEROPs protease and CAZyme families enriched in these ortholog clusters show significant copy number expansions within *Bsal* isolates [22], bolstering the hypothesis that gene duplication and subsequent sequence divergence and selection supports *Bsal* evolution. Should the *Bsal* epizootic continue, and the collection of isolates grow, then further studies could focus on linking individual genes to phenotypes. As many of the gene functions displaying signatures of positive selection appear to relate to pathogenicity, a key step in support of this could be the development of an *in vitro* model of infection as developed for *Bd* [46], enabling detailed description, as performed *in vitro* in this study, but detailing *in vivo* phenotypes.

Phenotypically, we observed variation in multiple lifecycle measurements, such as zoospore attachment, zoospore densities, fecundity, growth rates and lifecycle length. We found isolates displayed different responses in all lifecycle measures across the tested thermal gradient. Many of these responses may influence disease dynamics, for example isolates displayed higher fecundity either side of their optimal growth temperatures (i.e. highest fecundities are seen at 0°C, 4°C, and 20°C). This may correspond with observations in *Bd* where at cooler temperatures growth rates are slower but zoospores remain active for longer increasing zoospore density, contributing to higher zoospore encounter rates and supporting effective chytridiomycosis infection rates despite suboptimal growth [17]. These insights also have important implications for scientists working with *Bsal;* both for supporting successful *in vitro* work, but also as temperature-based regimes are the most common method for treating *Bsal* [25,47]. Indeed, it remains unknown how accurately *in vitro* observations represent *Bsal* responses *in vivo*, and an important next step is evaluating how such isolate variation across temperature gradients influences *Bsal* disease dynamics (and treatment efficacy) *in vivo*.

This work highlights the importance of evaluating how isolate variation across temperature gradients influences *Bsal* disease dynamics *in vivo*. One of the main challenges for continuing this work will be developing an appropriate model system [6,20,48]. All of the *Bsal* isolates included in this manuscript were associated with mass mortality events in their source population and rapid fatal chytridiomycosis under laboratory conditions in fire salamanders (*Salamandra salamandra*). Studies aiming to differentiate virulence between some of the *Bsal* isolates described here, performed in the highly sensitive *Salamandra* species, predict that the very high sensitivity of such individuals (experiencing fatal chytridiomycosis within weeks) leads to little potential variation in disease course such that large sample sizes would be required to tease apart these isolate differences (20+ animals per group, Fig W in S1 Text). While in such highly sensitive species and in the optimal thermal range for *Bsal*, these isolate differences may be subtle enough to be inconsequential in the context of the epidemic; as our data shows, across a wide thermal range and in less sensitive host species these dynamics are likely to play a significant role. Thus, developing an appropriate model system to elucidate the complex interactions of host, pathogen and temperature is an important but difficult next step, that fell beyond the scope of this work.

We observed isolates tolerant of both hotter and cooler temperatures than the BundBos2013 type strain, both of which help to explain observed population effects. First, isolates with thermal optima above 20°C corroborate *Bsal* prevalence in subtropical regions, at temperatures higher than tolerated by the type strain, in Vietnam [49] and the Ryukyu Archipelago [19]. Conversely, the indication that some isolates (e.g. Eifel2019) can grow well at lower temperatures with an optimal temperature for growth around 6°C could explain recent evidence suggesting ‘silent’ (deduced) mass die-offs in in the southern Eifel region [50]. Specifically, while BundBos2013 can survive the temperatures of overwintering salamanders, it would likely be reproducing at insufficient rates to effectively cause disease. However, it seems that some isolates may not only survive, but thrive at low winter temperatures. Given estimated fire salamander population declines of up to 90% within 6 months after *Bsal* introduction [20], if introduced in summer or autumn *Bsal* could spread rapidly within the adult population, aided by autumn mating [20] and winter aggregation [51,52] behaviours. Large numbers of individuals may then succumb to fatal chytridiomycosis over winter when these amphibians are largely inactive—such that no mass mortality event would be observed. This also provides an interesting example of the complexities of emerging infectious diseases. Specifically, while there is much discussion of the adaptation of fungi to raising temperatures with climate change and associated risks to mammalian species [53], globalisation simultaneously leads to the anthropogenic movement of pathogens from tropical to temperate regions, and the adaptation of such isolates to lower temperatures. The latter scenario may pose a particular risk to ectothermic hosts, such as amphibians [54], reptiles [55] and insects [56], whose immune defences are significantly reduced at low temperatures [56]. As the body temperatures of most amphibians closely follow microhabitat temperatures [24], temperate species may spend significant portions of the year (or indeed the entire year) in a state of elevated vulnerability.

We showed that *Bsal* can grow at a much larger range of temperatures than previously observed. This capacity also expands opportunities for growth across its potential invasive distribution. The variation in our predictions of thermal *Bsal* growth across European fire salamander populations support this notion and underline the continuous risks posed by new introduction events. Nevertheless, caution should be taken when interpreting these predictions. The *in vitro* relationships between temperature and pathogen growth which we show here may not necessarily represent those same relationships *in vivo* [57,58]. Our predictions therefore illustrate how peak growth of different *Bsal* isolates may vary across the fire salamander distribution range, but the maps are not directly useable to inform local management efforts. Improved predictions should be generated for this purpose once broad-scale, high-resolution environmental data become available. Nevertheless, there is little doubt that the similar microclimate conditions occupied by terrestrial salamanders across Europe enforce vulnerability to *Bsal* throughout the year, especially since different isolates peak at different temperatures within this narrow range. The relatively high thermal tolerance of several isolates may furthermore expand *Bsal*’s potential invasive range to include low-elevation, (sub)tropical urodele populations, for example in North, Central and South America, while isolates tolerant of cooler temperatures may threaten urodelan populations at high altitudes, for example in Central and South America.

Our work also highlighted an interesting phenotype, with isolates producing enlarged sporangia at 20°C (see supplementary discussion). This temperature falls beyond the range generally experienced by terrestrial northwest-European urodeles [24], but remains within the *Bsal* reproductive range. These sporangia are not associated with high productivity; in fact, their fecundity (number of zoospores/sporangia area) is lower. It is well established that for many dimorphic mammalian fungal pathogens, higher temperatures (approaching mammalian body temperatures) are a trigger to form the pathogenic phenotype. However, dimorphic fungal pathogens of ectotherms such as insects and plants are principally thought to react to chemical sensors to trigger morphing to a pathogenic form [59]. Unlike most insect and plant hosts, body temperatures of urodele amphibians generally follow relatively stable, cool, and moist microclimate conditions [24]. Accordingly, while clearly not truly dimorphic, the relative stability of the urodele thermal range may have supported *Bsal-*isolate adaptation to recognise temperatures outside of this normally restricted host tolerated range, but within its own thermal tolerance, and adopt a phenotype better suited to surviving in such conditions. As no *Bsal* isolates have been collected from East-Asia, we cannot know if this phenotype is observed in endemic populations at higher temperatures, or if it is associated with the absence of pathogenicity observed in those populations.

Furthermore, our analyses can only present a snapshot of the *Bsal* European epidemic. *Bsal* clearly has a vast capacity for rapidly evolving and adapting, and the diversity present in endemic Asian *Bsal* populations (and thus potential future introductions) is completely undescribed. Moreover, many elements of *Bsal* thermal ecology remain unknown, for example the impact of sequential introductions of isolates with different thermal curves. While perhaps different isolates could thrive in different seasons leading to a broadening of the period of vulnerability, conversely, perhaps isolate competition driven by other characteristics would lead to dominance of one strain over another or even hybridization. Understanding *Bsal* diversity, and how it can vary over time, would strengthen our ability to predict and mitigate future outbreaks.

As novel pathogens emerge at an accelerated pace, disease ecologists are increasingly aware of the impact of pathogen variation on disease dynamics. As divergent variants may respond differently to a plethora of host, biotic and abiotic factors, teasing apart the role of pathogen variation in population level disease dynamics is extremely complicated. However, such insights are particularly important in emerging infectious diseases, where entire host populations may be immunologically naïve and highly susceptible to the pathogen, such that even small changes in virulence or host-pathogen dynamics can have large consequences on the population-scale. To better understand, predict and mitigate amphibian losses due to the emergent *Bsal* fungus, we need to better understand what variation is present and how environmental factors influence the biology of this pathogen.

## Supporting information

S1 Text Word document containing all supplementary discussion, figures and tables. (DOCX)

S1 Data Excel sheets of GO Term Enrichments and MEROPs or CAZYme annotations of groups of genes with the largest dN/dS values and dN/dS values greater than 1. (XLSX)
