# Supplementary material for: High phenotypic diversity correlated with genomic variation across the European Batrachochytrium salamandrivorans epizootic
Source: PLoS Pathog. 2024 Oct 16;20(10):e1012579. doi: 10.1371/journal.ppat.1012579 (PMC11515996; doi:10.1371/journal.ppat.1012579)
Supplement: S1 Text — (DOCX) [file ppat.1012579.s001.docx]

### S1 Text:

### Supplementary Information:

**High phenotypic diversity correlated with genomic variation across the European *Batrachochytrium salamandrivorans* epizootic**

**Text A: Supplementary Discussion**

We also observed a yet-undescribed phenotype produced at 20°C, consisting of exceptionally large sporangia with an area 3+ times larger than the area of sporangia seen at other temperatures (Fig X). While enlarged sporangia were particularly pronounced in some isolates, such as Essen2019, all isolates seemed to produce larger sporangia at 20°C and displayed increased mean and maximum sporangia sizes (Fig Y). As seen circled in Fig 3B in the Main Text, in Essen2019 the presence of enlarged sporangia was observed with marginally higher qPCR values compared to wells from the same isolate and temperature without these sporangia. However, as fewer enlarged sporangia were produced, sporangia coverage at 20°C remained similar to, or lower than, that at 15°C (Fig Y) and they weren’t associated with significantly better culture growth in terms of qPCR score, total (zoospore and zoosporangia) counts or total sporangia coverage.

**Supplementary Figures**


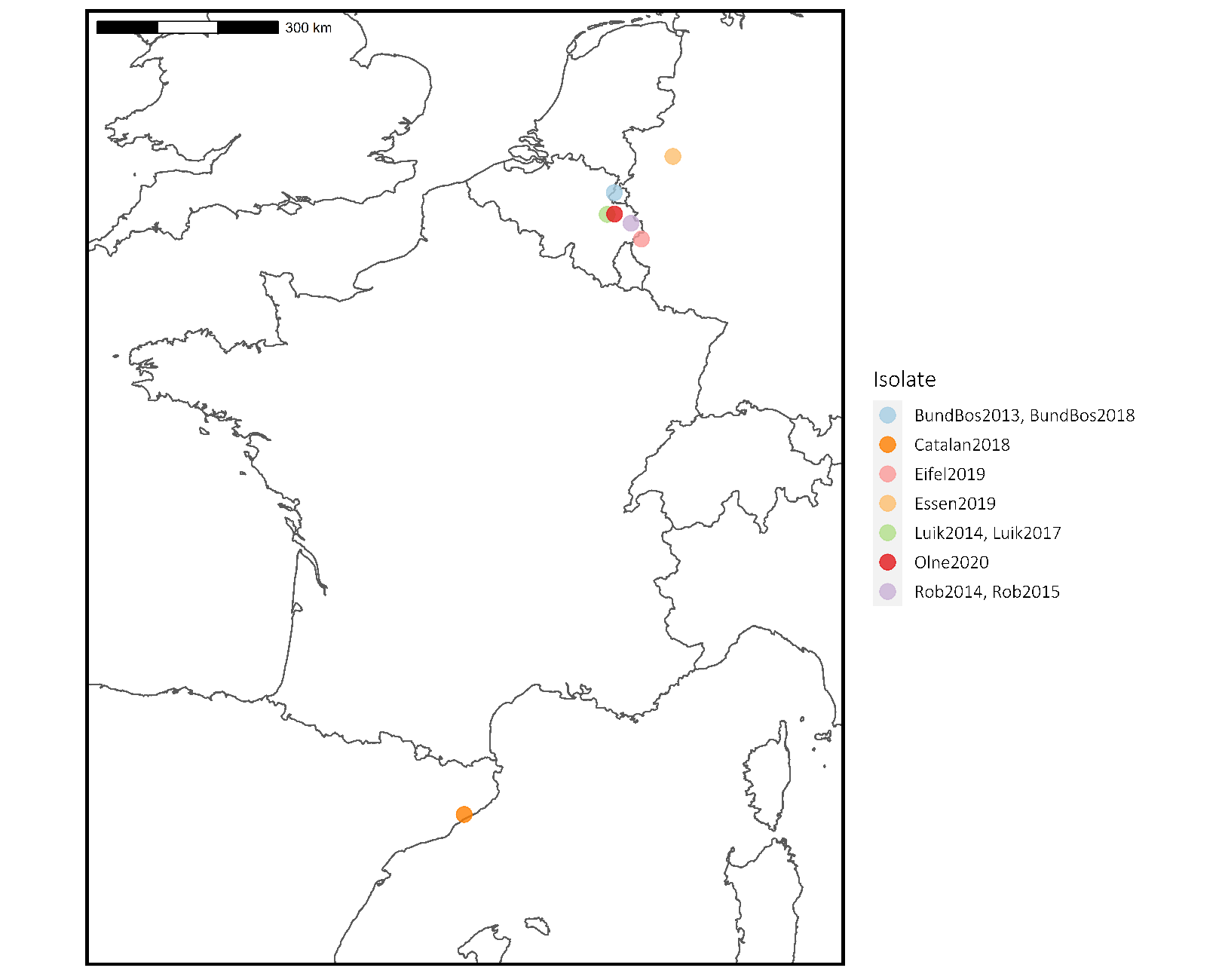


Fig A. Map with locations of outbreak sources of isolates. Locations of captive isolates are not provided to maintain anonymity. Generated using countries geojson file from <https://api.datahub.io/core/geo-countries>


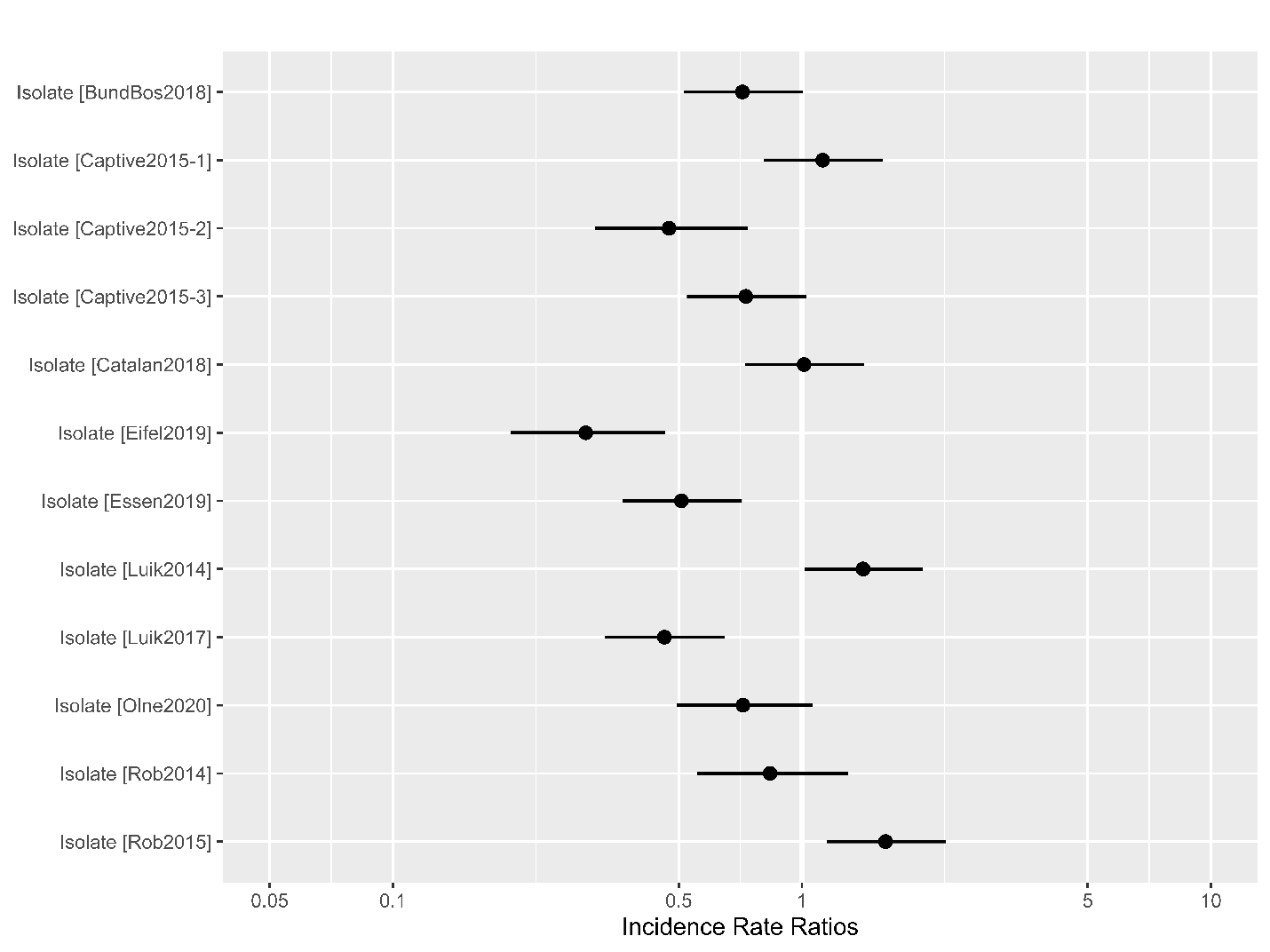


Fig B. Zoospore attachment variation. Incidence rate ratios, with 95% confidence intervals, from mixed effect model with negative binomial distribution, with structure spore count ~ isolate (Observations = 282, Marginal R2 = 0.213), with BundBos2013 isolate as intercept- with IRR = 85.50, 95% CI = 69.48 – 105.22. See Table G for post hoc pairwise comparisons.


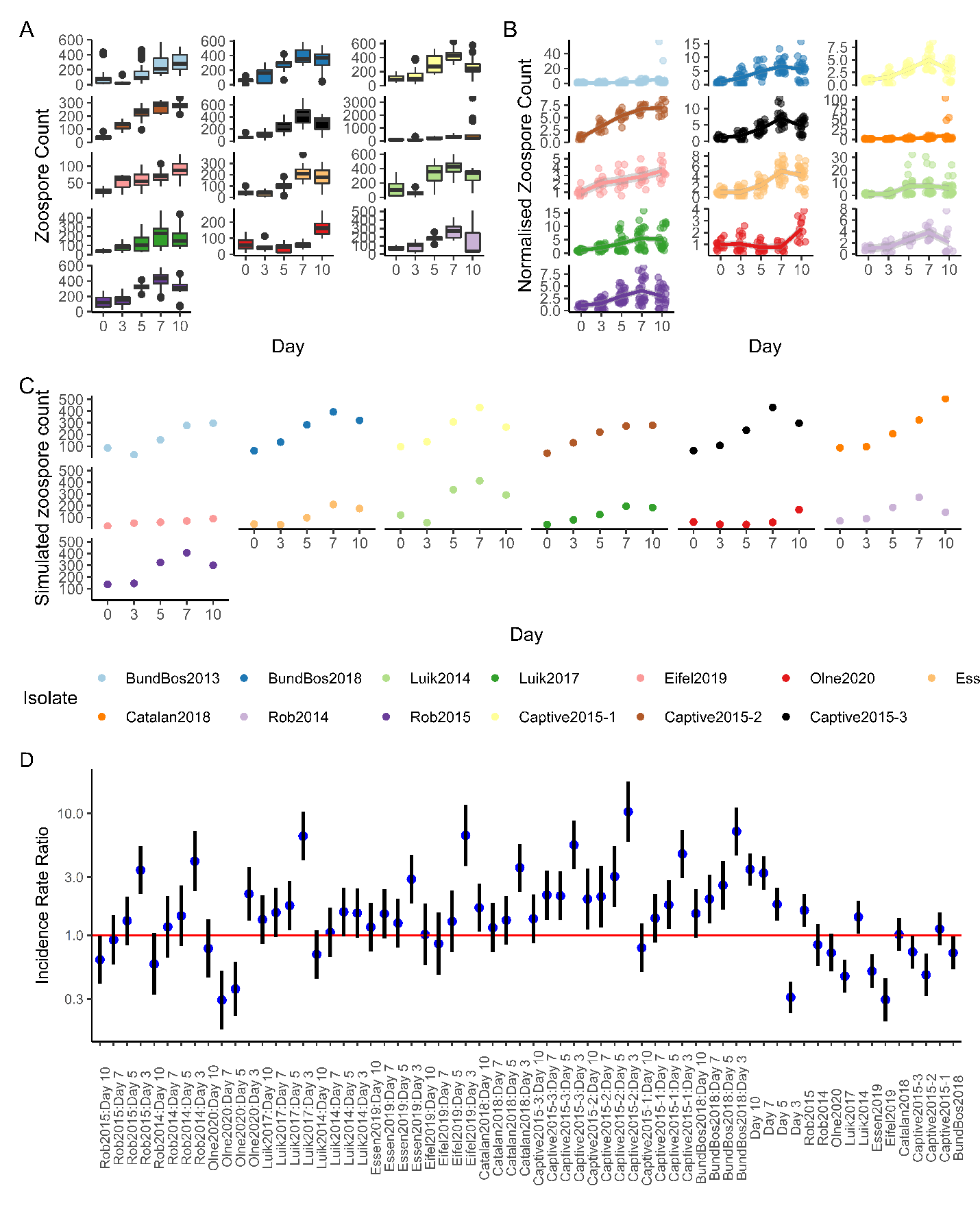


Fig C. Variation in zoospore counts. A) boxplots of zoospore counts of all isolates over time at 15C. B) zoospore counts per isolate over time standardized by initial spore attachment by dividing by spore count at T=0. C) simulated data from of negative binomial model, accounting for observer, indicates relationship of each isolate over time. D) incidence rate ratios, with 95% confidence intervals, from a glm with a negative binomial distribution with structure zoospore count ~ isolate*day, treating time as a discrete variable due to non-linear relationship with time (Observations = 1353, R^2^ = 0.849), with BundBos2013 isolate as intercept- with IRR = 85.5, 95% CI = 71.1-103.9, p <0.001).


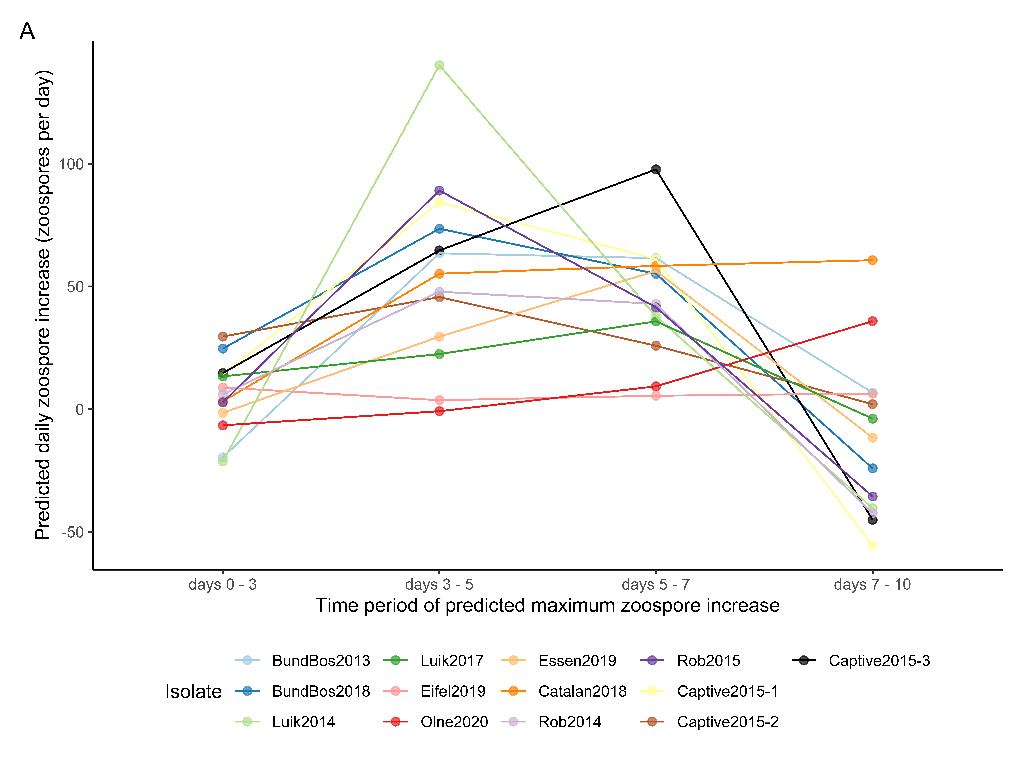


| B  Isolate | Predicted sporulation time period (day) | Predicted zoospore increase (daily) |
| --- | --- | --- |
| BundBos2013 | days 3 - 5 | 63.5 |
| BundBos2018 | days 3 - 5 | 73.5 |
| Captive2015-1 | days 3 - 5 | 84.3 |
| Captive2015-2 | days 3 - 5 | 45.7 |
| Captive2015-3 | days 5 - 7 | 97.7 |
| Catalan2018 | days 7 - 10 | 60.7 |
| Eifel2019 | days 0 - 3 | 8.81 |
| Essen2019 | days 5 - 7 | 56.3 |
| Luik2014 | days 3 - 5 | 140 |
| Luik2017 | days 5 - 7 | 35.8 |
| Olne2020 | days 7 - 10 | 35.9 |
| Rob2014 | days 3 - 5 | 47.9 |
| Rob2015 | days 3 - 5 | 89.1 |

Fig D. Predicted sporulation timing from T0 of experiment- calculated by simulating data from generalized linear model (glm) with negative binomial distribution with formula zoospore count ~ isolate*day, with day treated as a factor due to non-linear relationship of spore count, the period with the steepest daily increase in spore count was then identified as the predicted period of sporulation


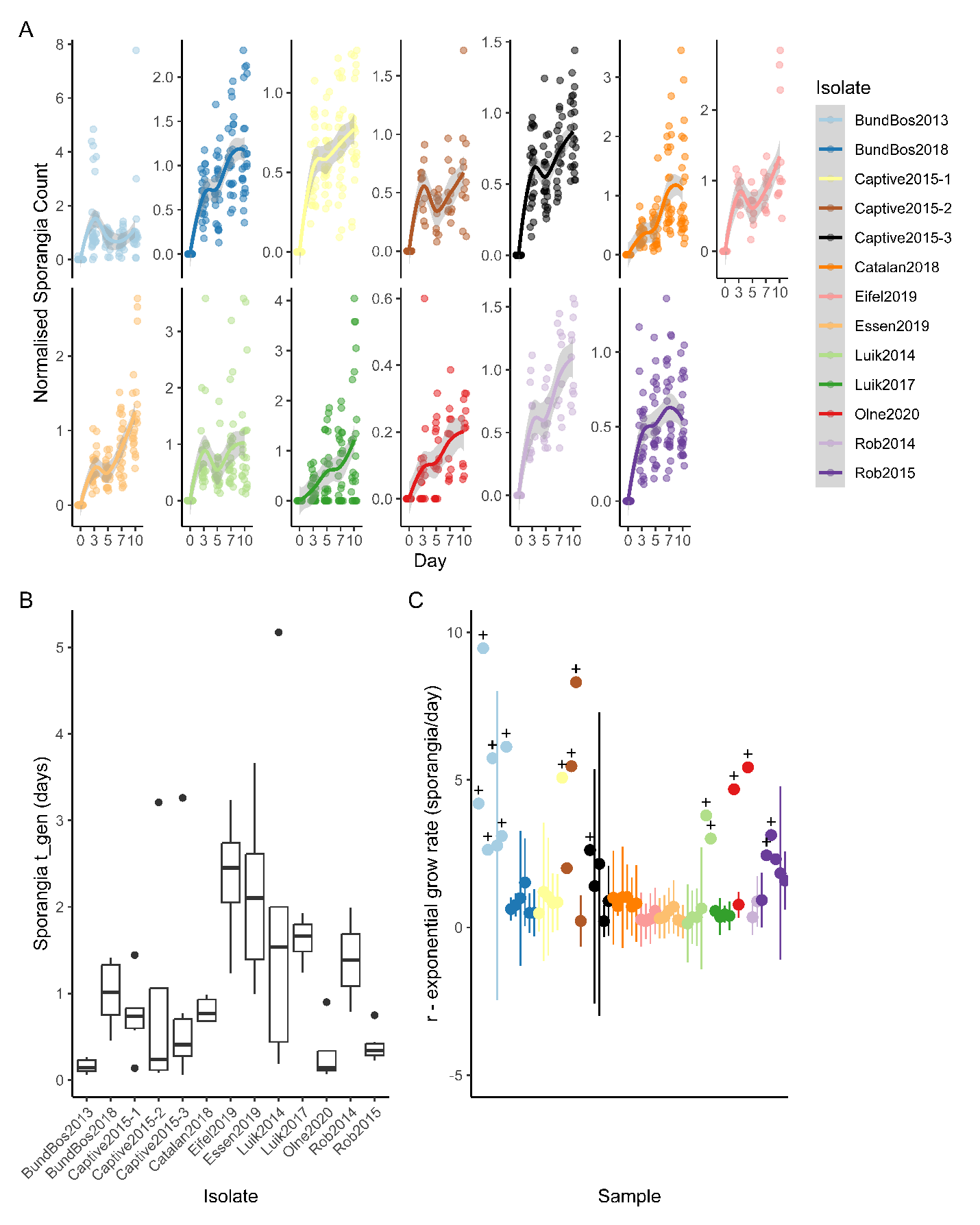


Fig E. Sporangia counts and sporangia count based growth curves at 15°C. A) Average curves of sporangia counts for all isolates, generated using loess function. B) boxplots of sporangia counts standardized by initial spore count (calculated sporangia count/average well spore count day 0) over time per isolate with smoothed average line (isolate colour) and 95% CI intervals (grey) calculated using the loess function. C) boxplots of generation times (t_gen), extracted from GrowthCurver growth curves constructed for each well, here higher numbers suggested a longer generation time so slower growth. D) Plot of growth rates, r, for each well – each point represents a well, points represent r value estimates, lines represent 95% CI, “+” denotes samples standard errors > 5 , for which confidence intervals aren’t depicted but can be visualised with the supplementary code.


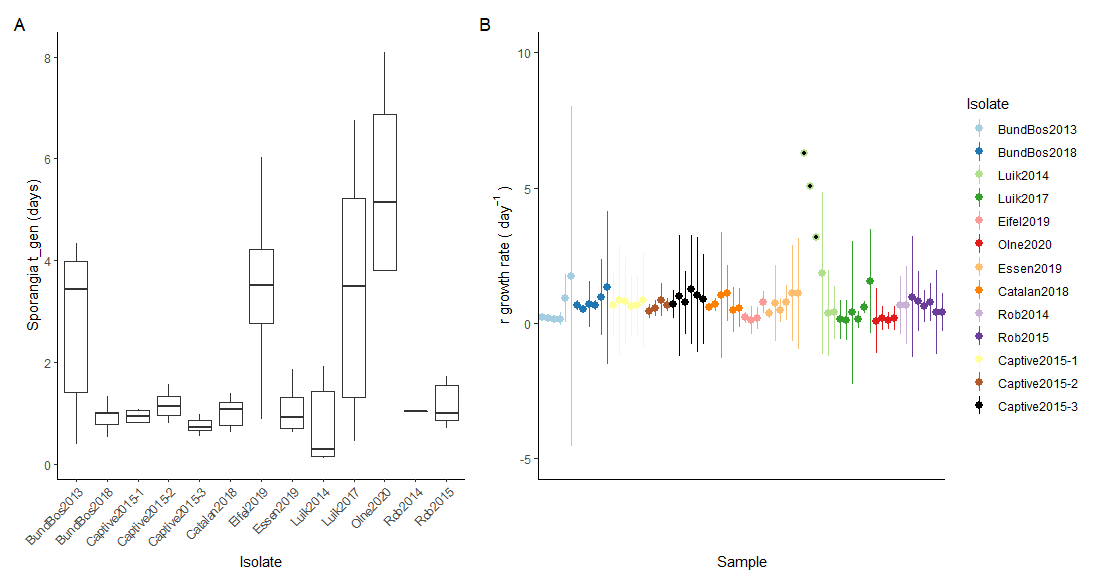


Fig F. Total (sporangia + zoospore) count based growth curves at 15°C. A) boxplots of generation times (t_gen), extracted from GrowthCurver growth curves constructed for each well, here higher numbers suggested a longer generation time so slower growth. B) Plot of growth rates, r, for each well – each point represents a well, points represent r value estimates, lines represent 95% CI, some samples had very high standard errors, the confidence intervals of these were not shown (but can be visualised from the raw data and code), these points are demarked by a black spot over the r value estimate point.


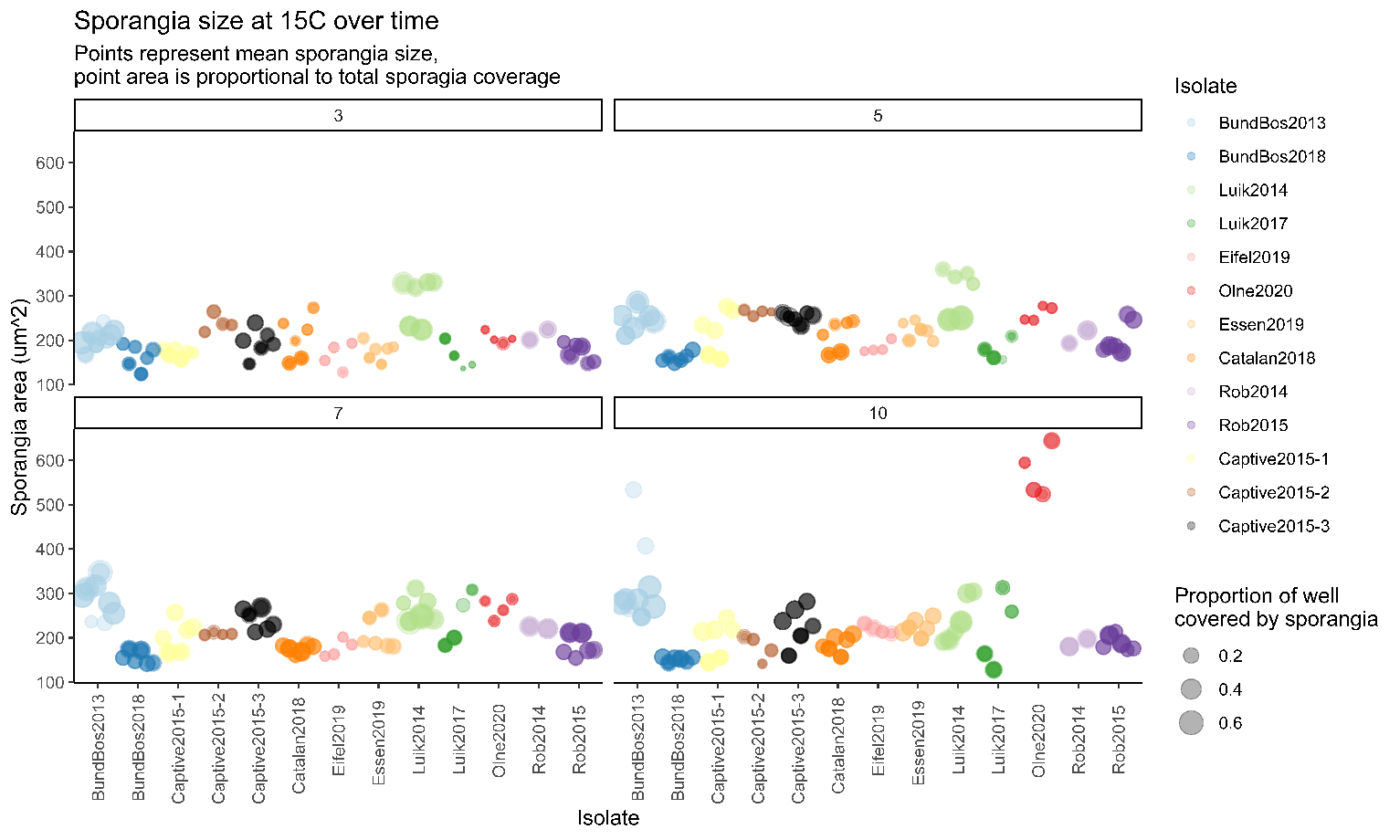


Fig G. Scatter plot of Sporangia size of different isolates over time. Point area size is representative of proportion of well area covered by sporangia, point location indicates mean sporangia size, lines indicated 95% CI of mean sporangia size.

^
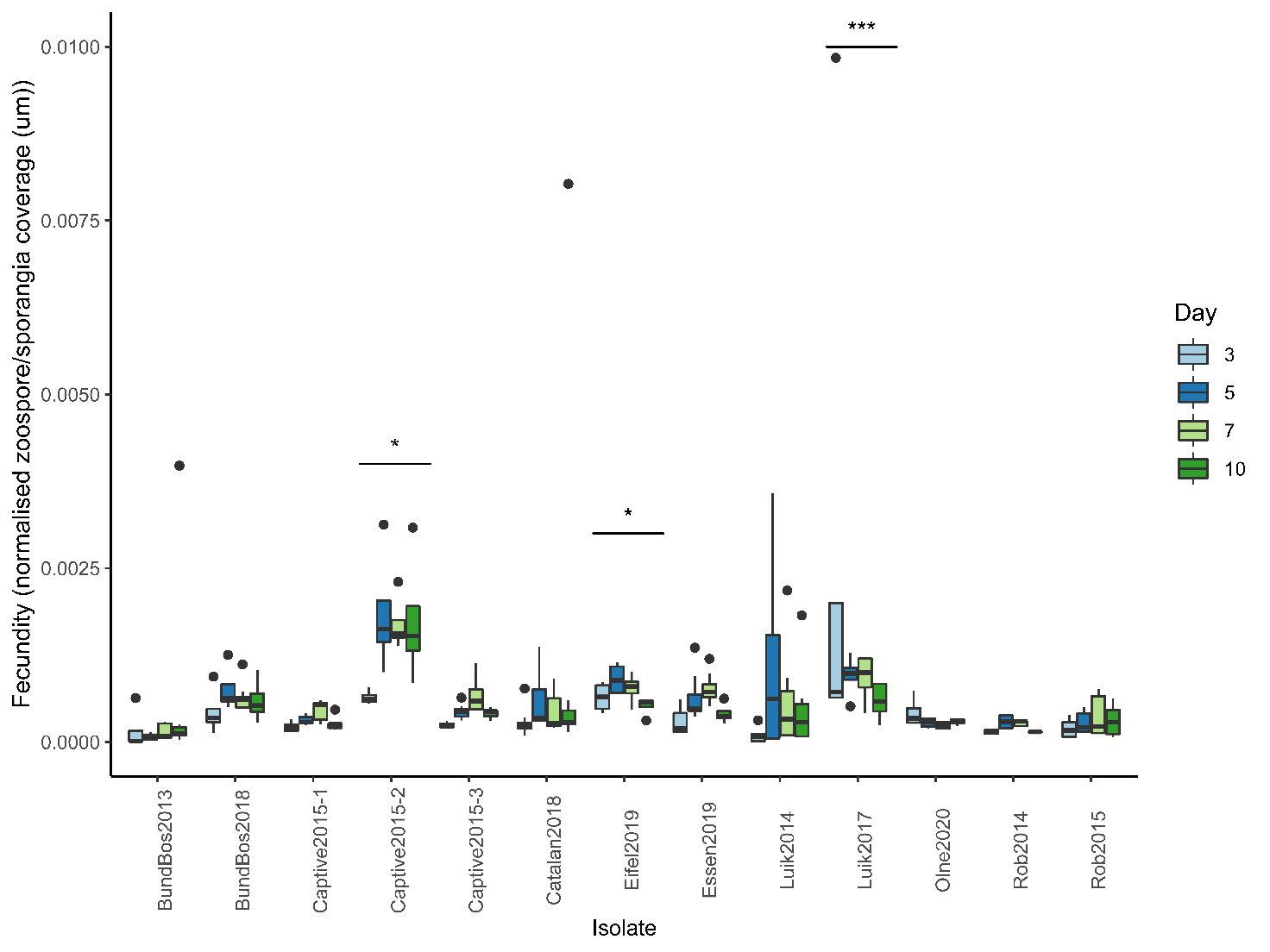
^

*Fig H. Boxplot of fecundity over time.* Boxplots show fecundity (standardized zoospore counts/zoosporangia coverage), over time. Zoospore counts were standardized to account for initial zoospore attachment by dividing zoospore count by average zoospore count of that well at t=0. Asterisks indicate isolates with significantly higher fecundity according to model glm with structure standardized fecundity ~ Isolate * as.factor(day), R^2^=0.29. see Table D for model details.


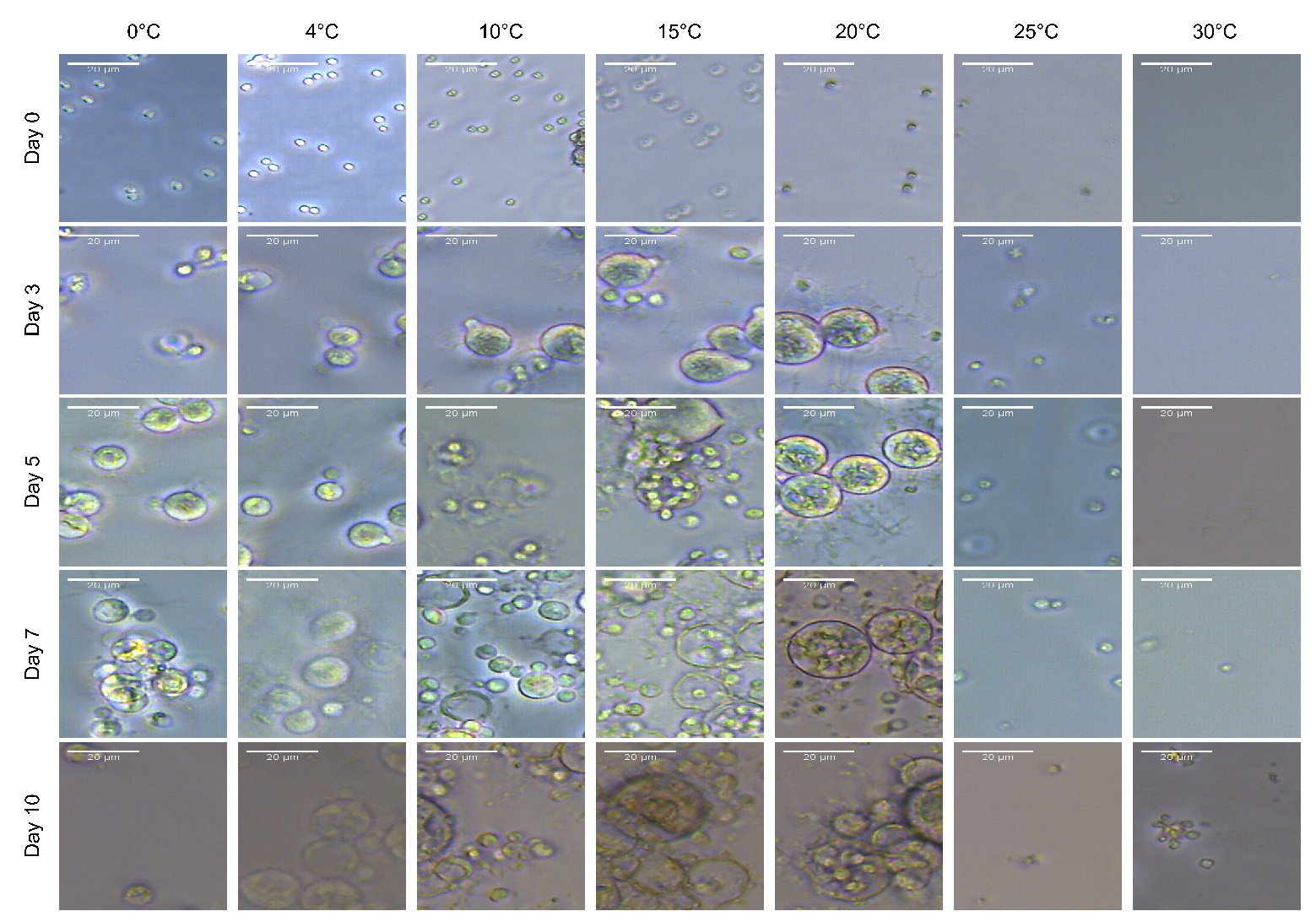


Fig I. representative images of BundBos2013 at all temperature and time points, photographed at 20x magnification, scale bars represent 20µm


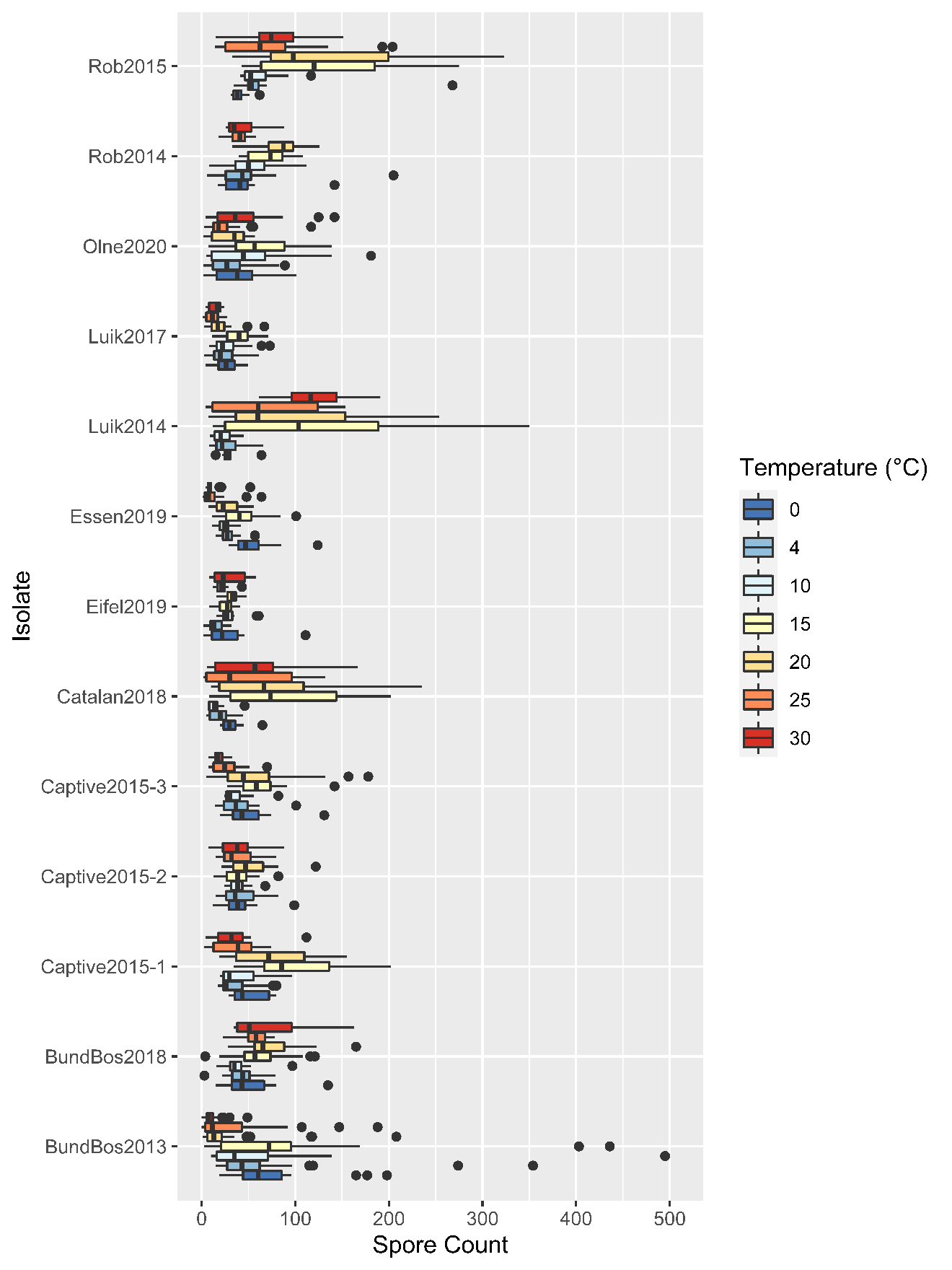


Fig J. Boxplots of spore counts at t=0 at various temperatures. This indicates spore attachment in the first 3 hours of incubation at the listed temperature.


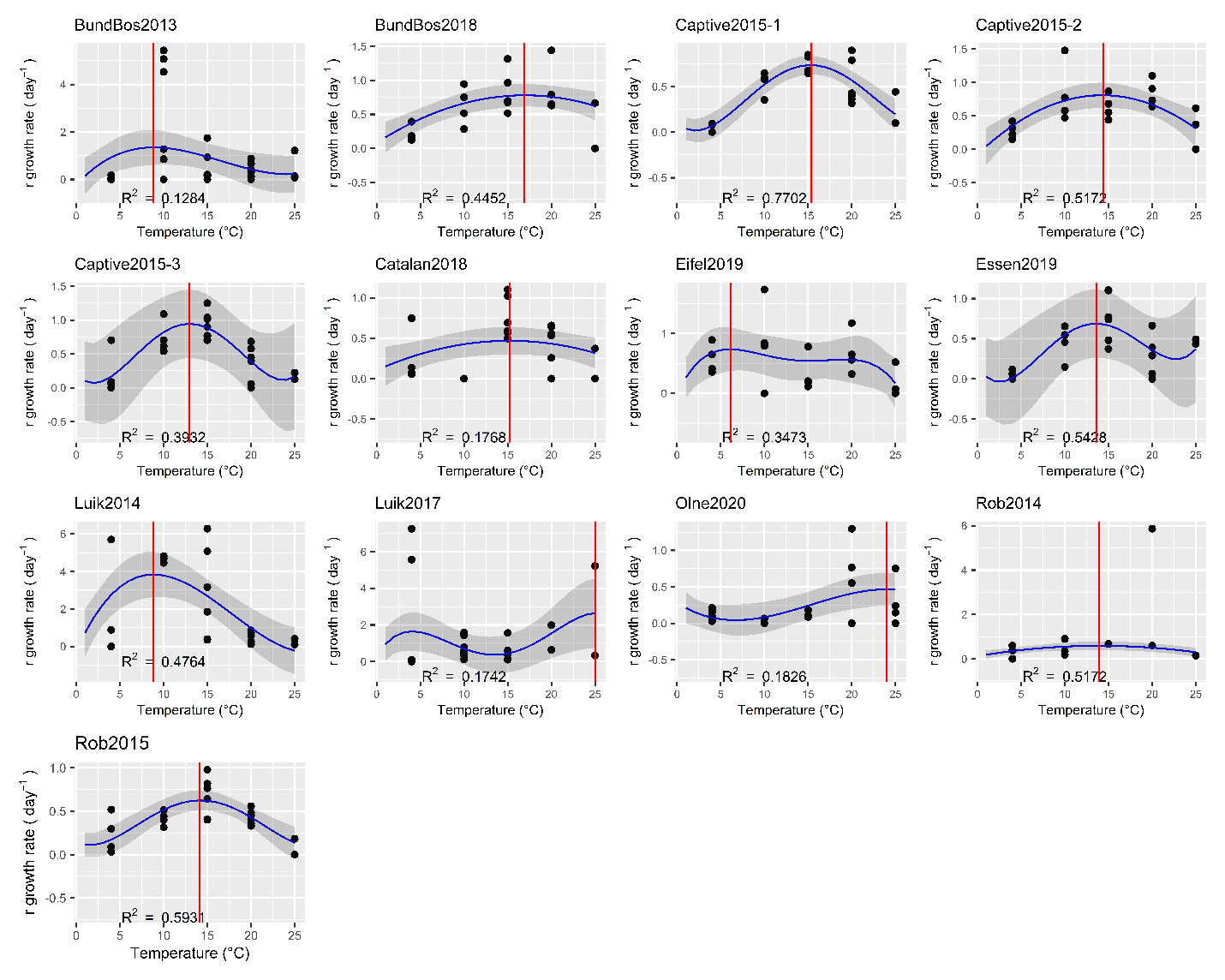


*Fig K. Isolate growth at various temperature regimes.* Optimal polynomials fit to growth rates (r value (count.day^-1^), calculated by GrowthCurver considering total (spore + sporangia) counts) vs temperature data (blue lines). with 95% confidence intervals (grey curves), red line indicates optimal temperature for growth, black dots represent raw r values, the R^2^ value for the fit of the polynomial to r growth rates is listed per each isolate.


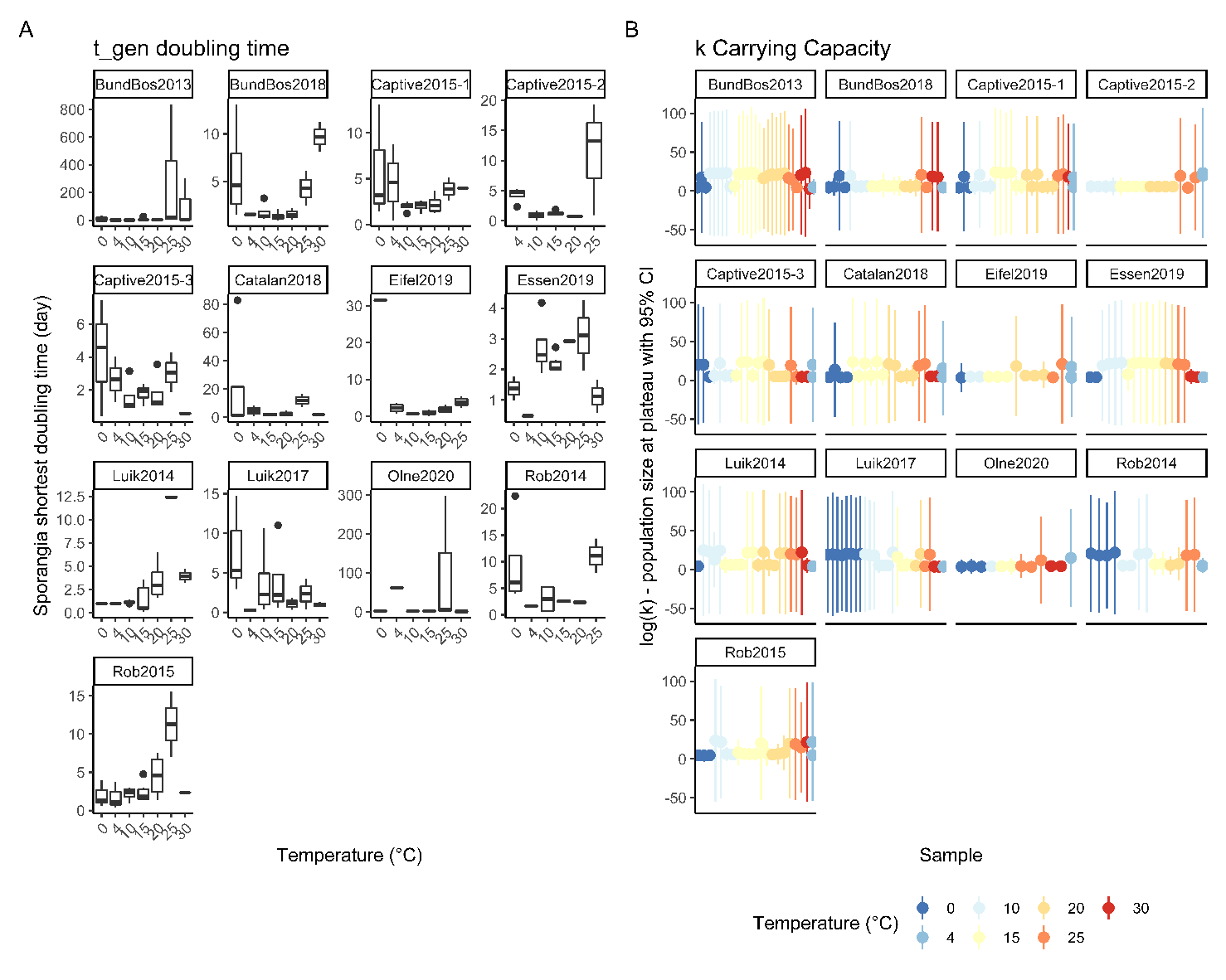


Fig L. Isolate outputs from GrowthCurver growth curves based on total counts (zoospore + zoosporangia counts), per well across a temperature gradient. A) boxplots of generation times (t_gen), extracted from GrowthCurver growth curves constructed for each well, here higher numbers suggested a longer generation time so slower growth. B) Plot of carrying capacities, k, for each well – each point represents the estimated carrying capacity of a well, lines represent 95% CI (mean +/- 2SE), colour denotes incubation temperature.


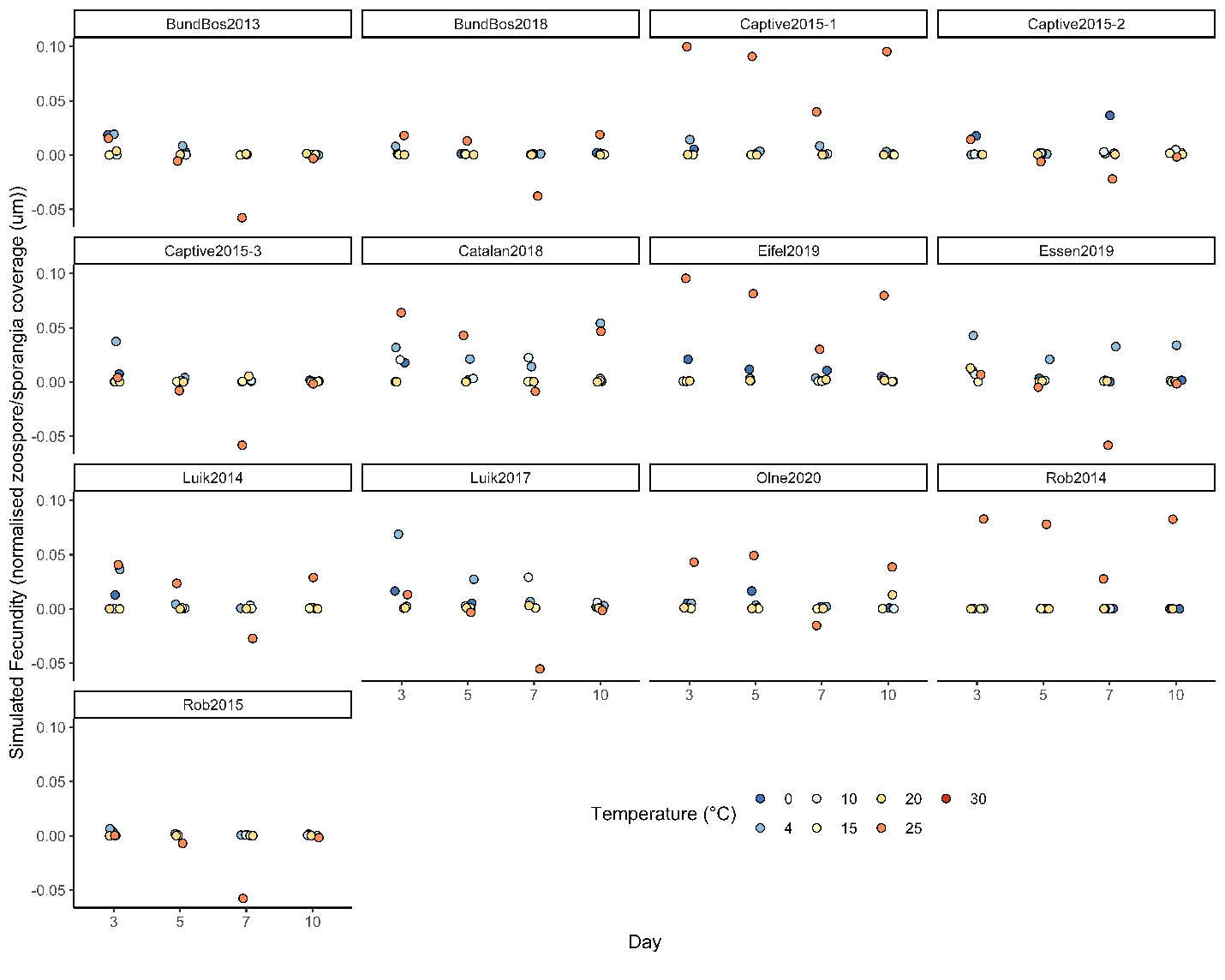


Fig M. Simulated Fecundity, standardized by initial spore attachment, across a temperature gradient, predicted using a glm (formula normalized fecundity ~ Isolate*as.factor(day)*as.factor(temp), Cox-Snell and Pearson pseudo R^2^ values =0.85), as fecundity did not show a linear relationship with either temperature or day both of these parameters are treated as categorical factors.


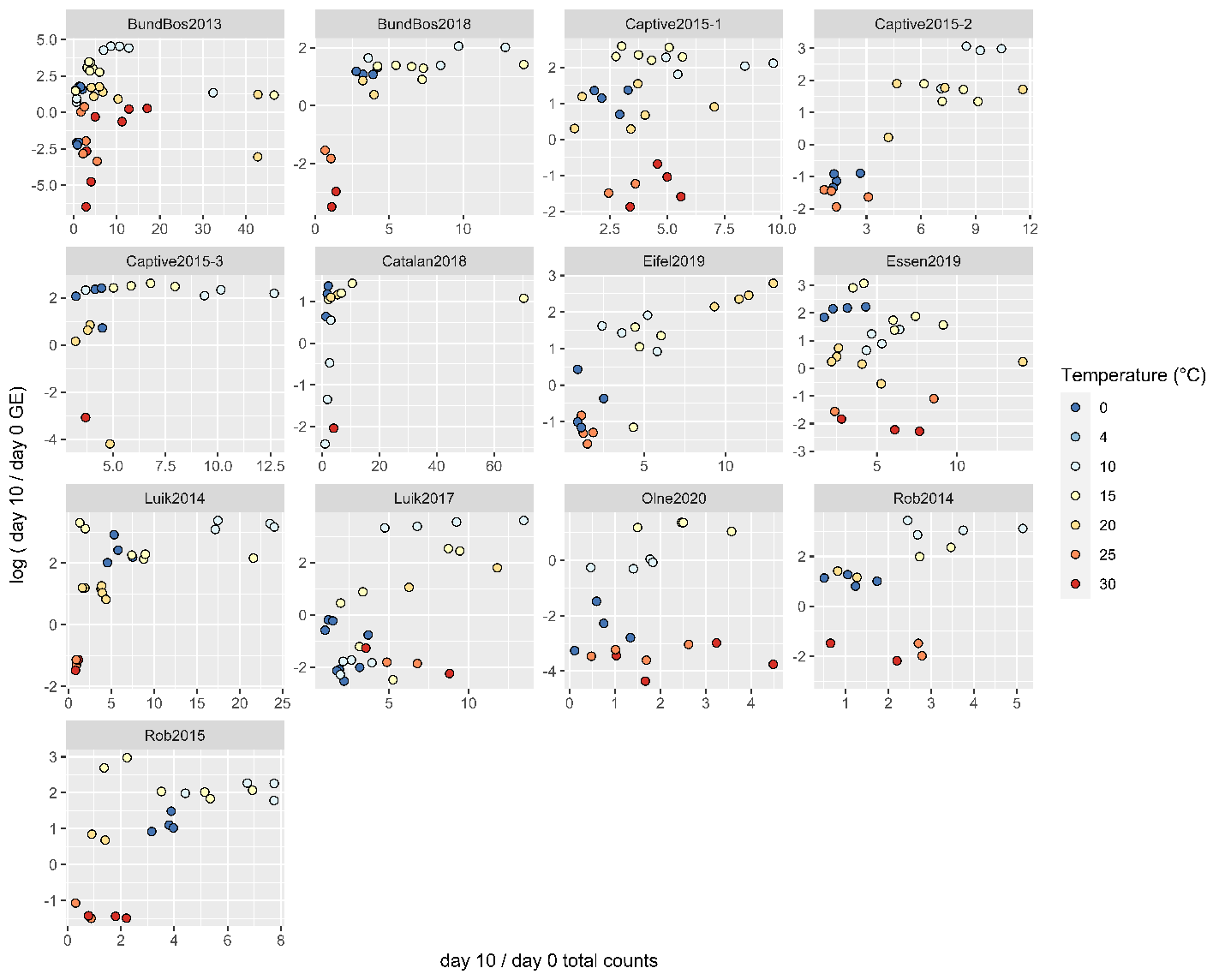


Fig N. Scatterplots of ratio of change in total counts from day 0 to day 10 vs log of ratio of change of genomic equivalents detected in qPCR. The change in total counts from day 0-10 is predictive of the GE change in this period (coefficient = 0.899, p = 2.66e-16, R^2^ = 0.18). A number of factors likely contribute to the low R^2^  – while total counts are specific per well, the qPCR tests are completed on different wells, also for total counts a full mature sporangia will count as 1 individual, but if about to sporulate this could contain numerous Genomic Equivalents.


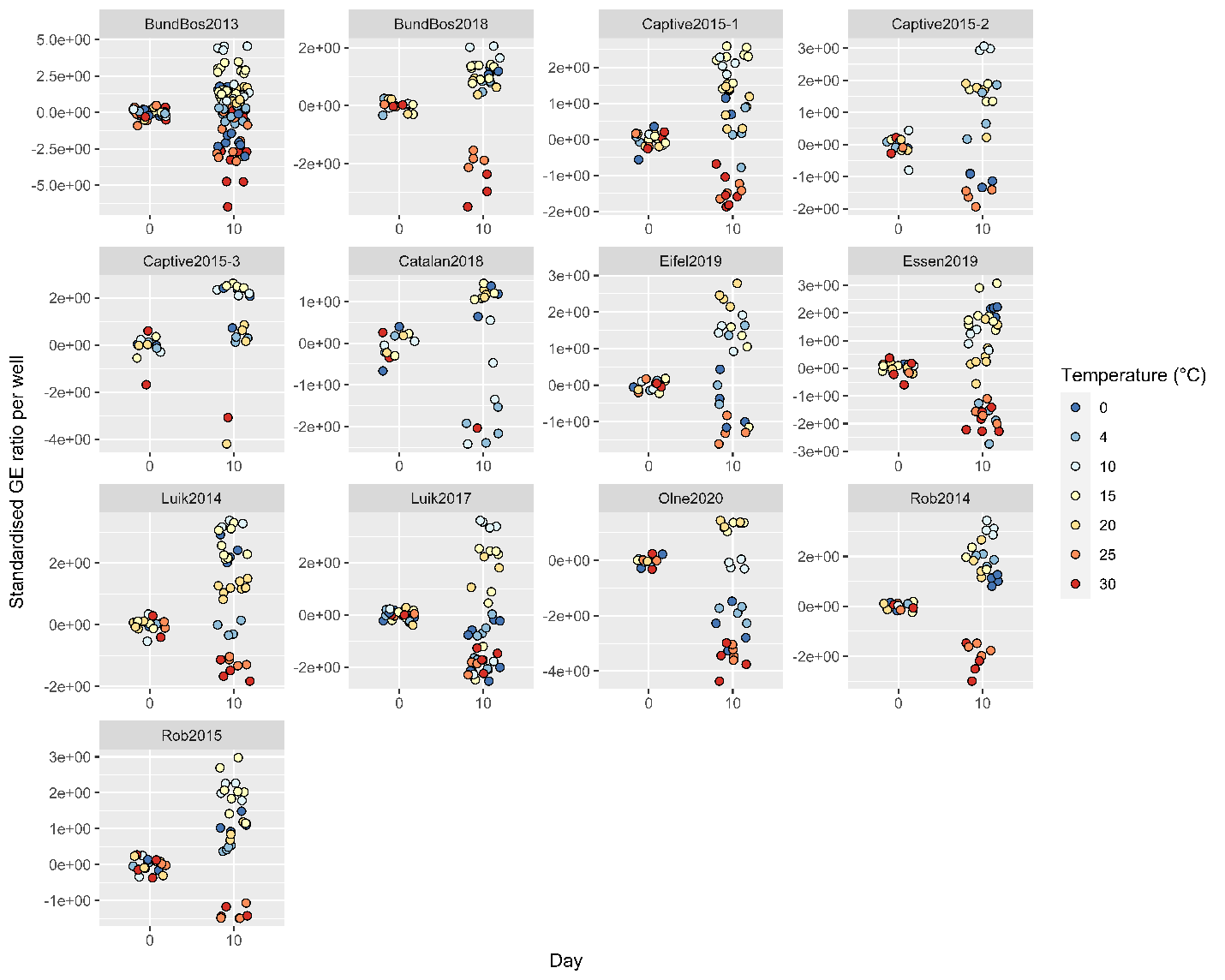


Fig O. Comparison of qPCR GE loads at day 10 and day 0, both normalized to average GE load at day 0 for well incubated at that temperature to account for differential zoospore attachment, at varying temperatures.


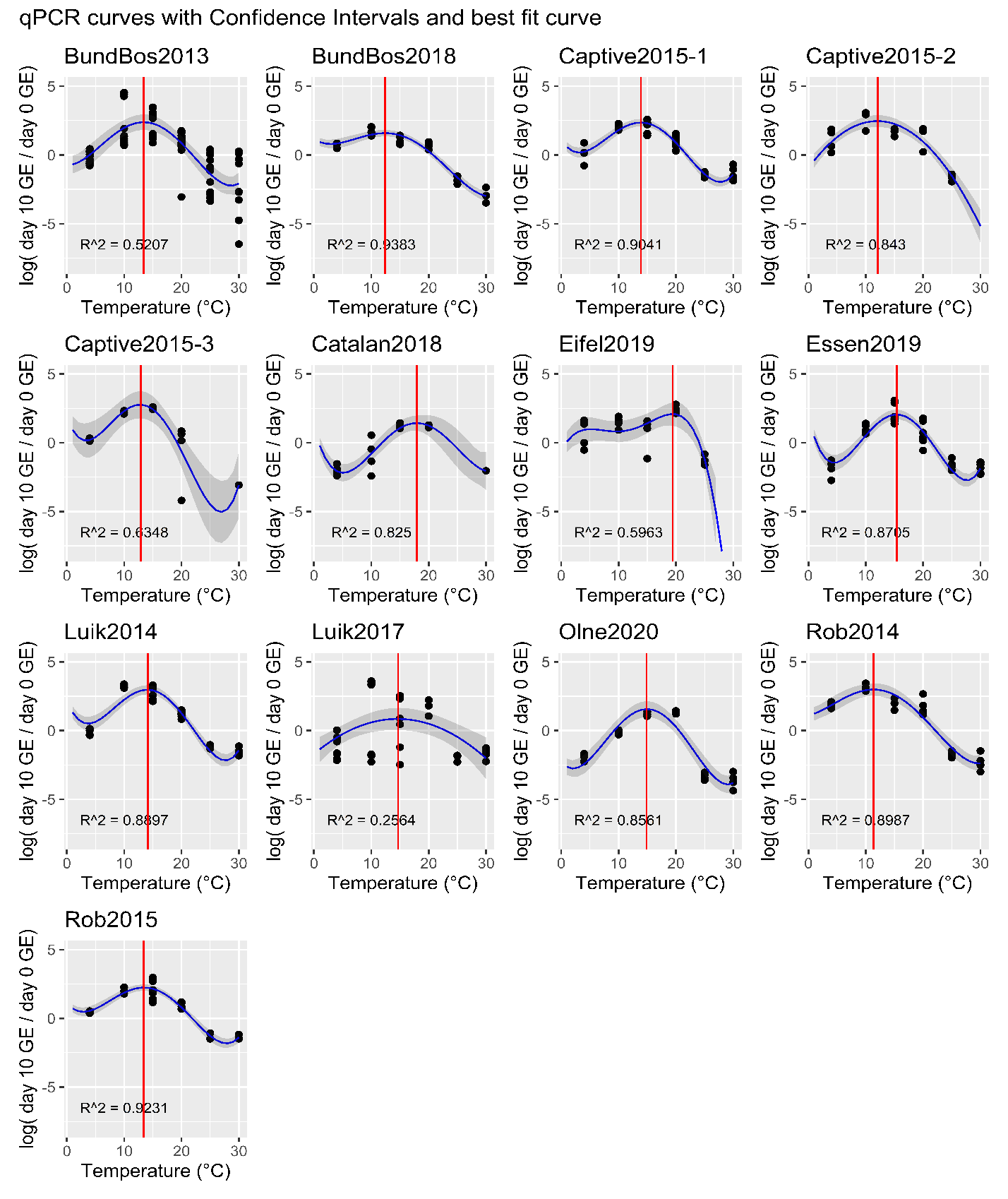


Fig P. optimal thermal performance curves based on qPCR results. Curves of log(GE ratio day 10/day 0) also show significant variation with optimal temperatures ranging >10 degrees. Curves are flatter than for GrowthCurver measurements- but may be influenced by numerous wells being visibly full, with some predicted to have reached carrying capacity as early as days 3-5.


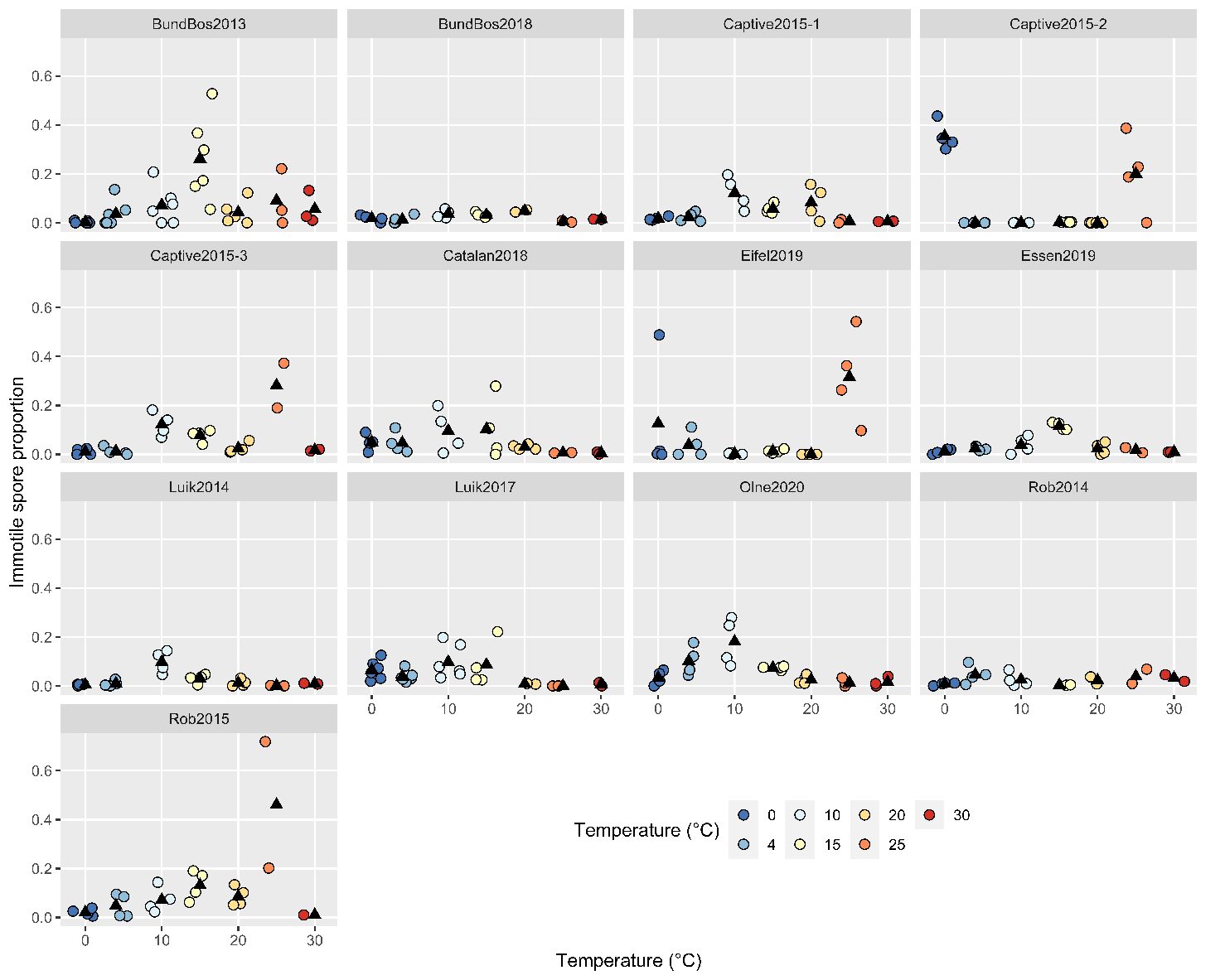


Fig Q. Proportion of spores produced as immotile spores. Scatterplot of proportion of spore count representing floating, immotile spores. Coloured circles represent raw proportions (per image), black triangles represent mean proportion per temperature and isolate.


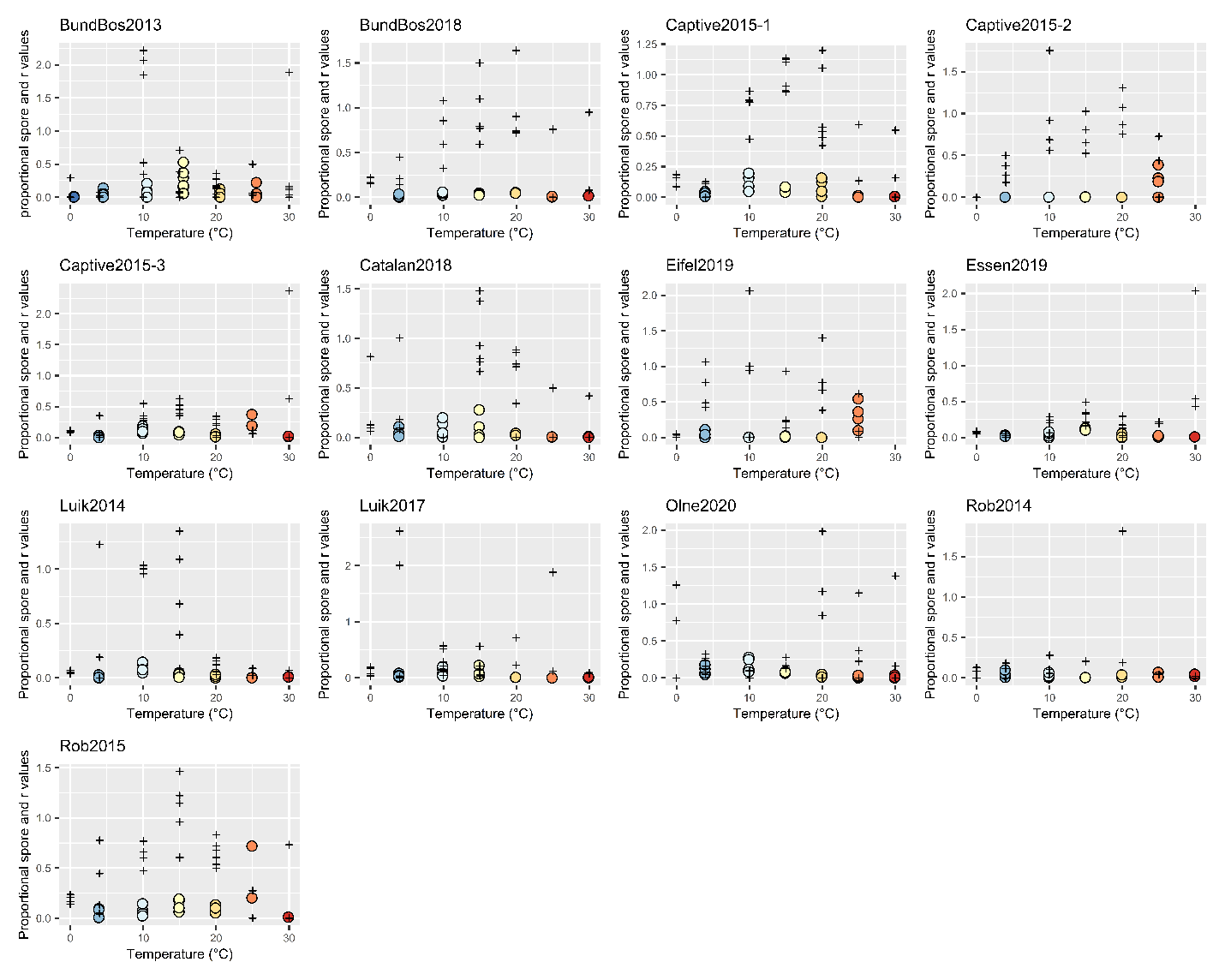


Fig R. Comparing standardized growth rates, r, with proportion of spores that are immotile spores. Given that it was hypothesized that such immotile spores, that are more resistant and long-living, would be preferentially produced in “tougher” conditions, we looked for associations between the growth rate, r (black crosses (+), calculated from growth curves fit by GrowthCurver to total (spore and sporangia) counts, standardized to bring onto a proportional scale as the proportion of immotile spores, by first calculating the average r value for each isolate at each temperature, identifying the maximum average r value for each isolate and dividing the extracted r values by this maximum average r value), and the proportion of spores produced that were immotile, resistant spores (coloured points- colour denotes incubation temperature).


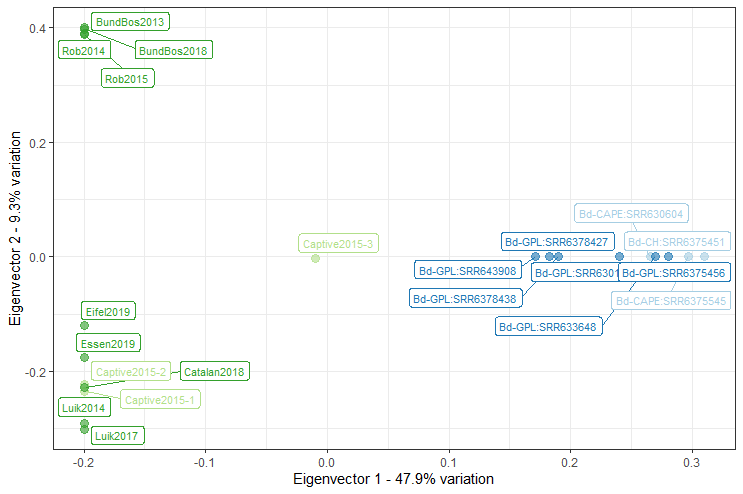


**B**

**A**


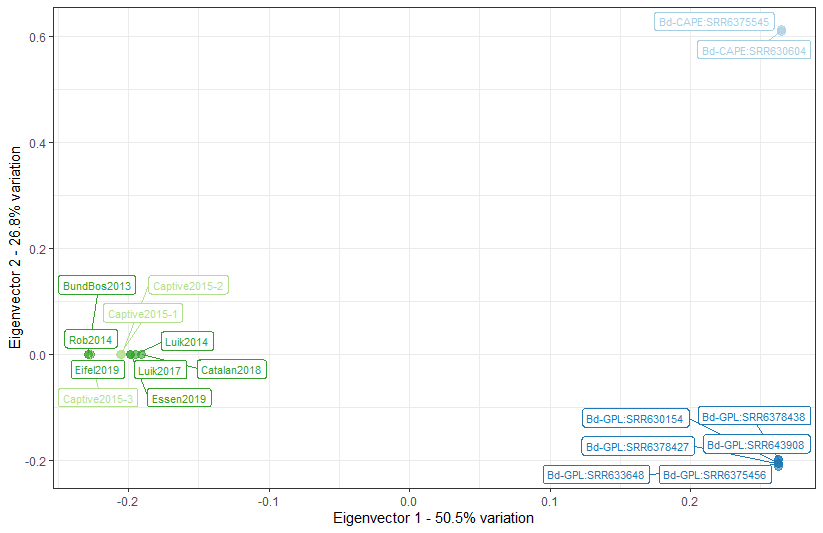


*Fig S. PCA of SNP variants called on all Bsal isolates and select Bd isolates aligned to A) BundBos2013 assembly B)* BdJel423 *assembly, calculated by snpgdsPCA() of SNPrelate.*

*
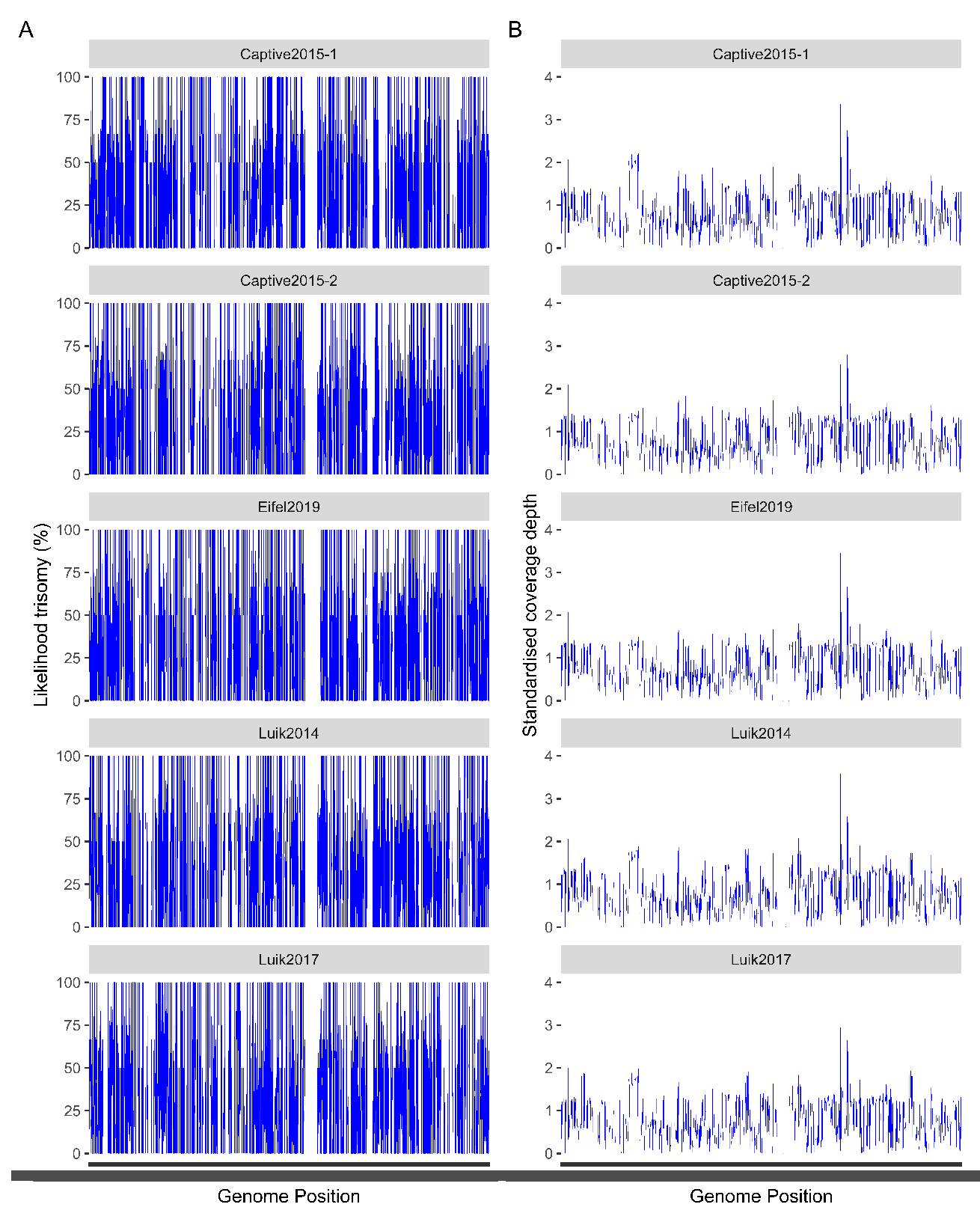
*

*Fig T. Assessments of ploidy in 5* Bsal *isolates, with reads aligned to the GCA_002006685.2 Bundbos2013 assembly.* Abundant bi- or tetra-allelic heterozygous SNPs were identified throughout the assembly, suggesting diploidy. A) We also identified high numbers of positions with tri-allelic probabilities, with the blue line indicating the proportion of heterozygous sites within 5kbp windows estimated to be tri-allelic. B) Illumina read coverage, standardised by the mean coverage of the largest contig, LYON02000001.1.


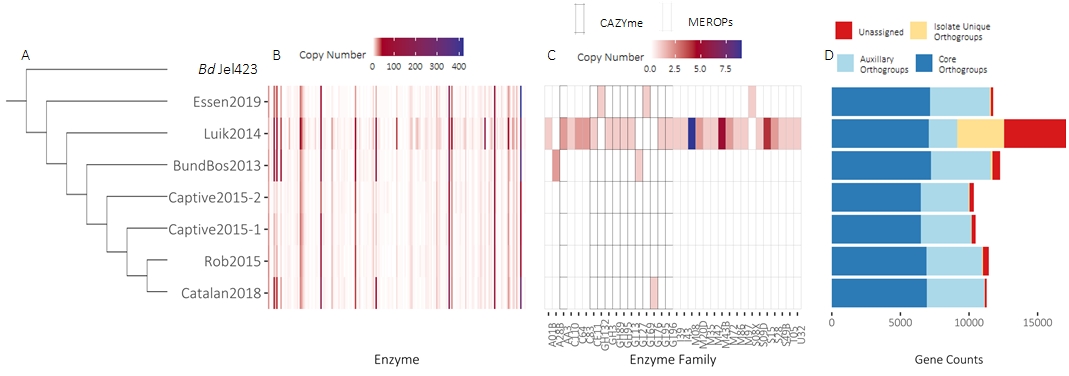


Fig U. Protein variation of Bsal assemblies. A) Protein based phylogeny, inferred using single copy core orthologs with OrthoFinder, B) Heat map of all candidate MEROPs and CAZYme enzymes C) heat map of CAZyme and MEROPs families with candidates present in only one isolate D) Bar chart of proteins identitified by OrthoFinder as belonging to orthogroups that are: dark blue = core to all isolates, light blue = auxiliary- present in 2+ isolates, yellow – unique to a single isolate, red = unassigned to an orthogroup- no orthologs identified in that or other assemblies. Adding the Essen2019 isolate, we saw this isolate sat within the genomic variation observed in the previously published Bsal assemblies. Interestingly, Essen2019 lack many of the genes associated with saprotrophic capacity described in Kelly et al. (2021[1]), but did contain candidates for two CAZyme families (GH132 and GT69), not described in the other Bsal isolates are associated with fungal cell wall function[2,3], and one serine protease family not described in the other Bsal isolates that may be associated with saprotrophic growth[4].

*
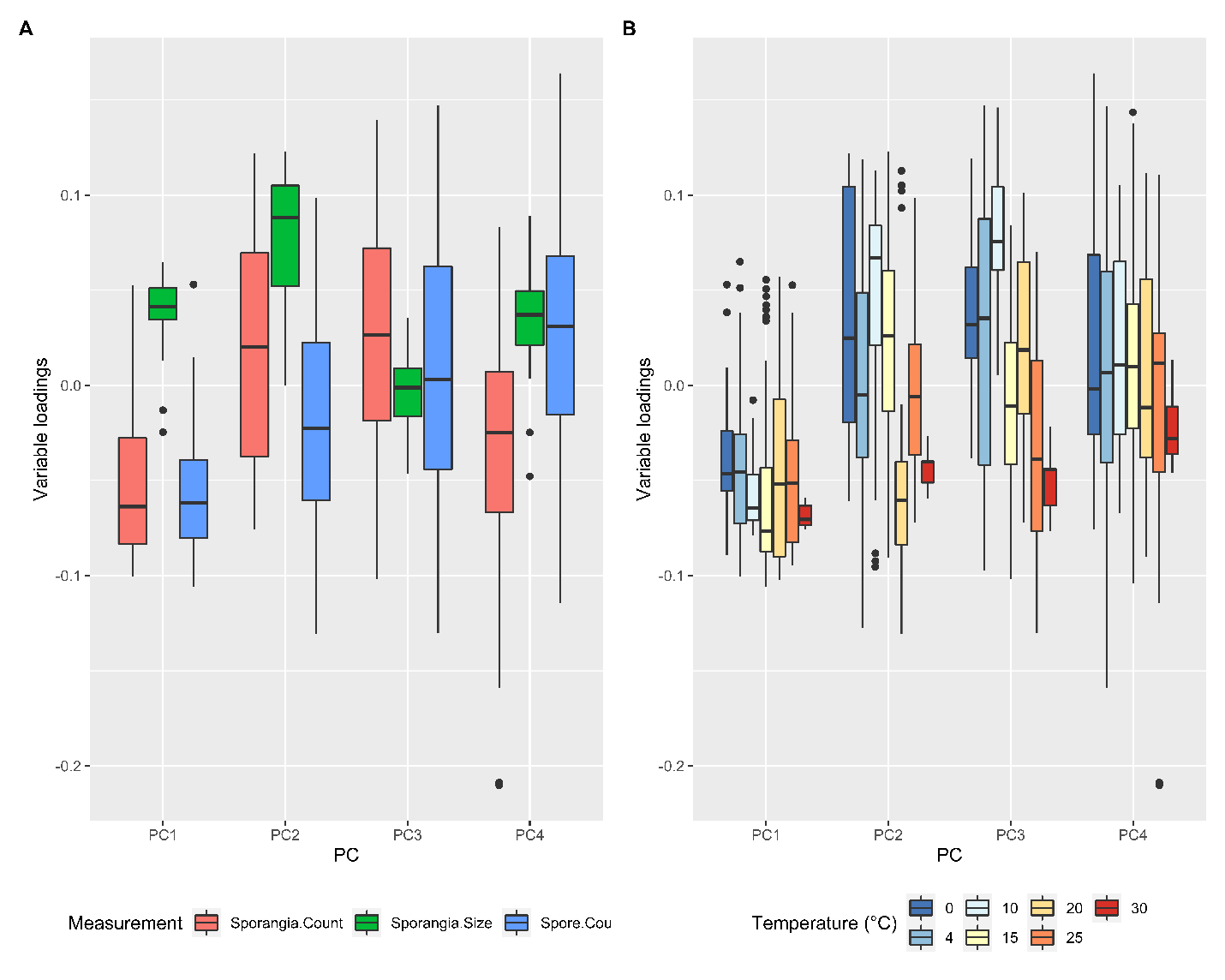
*

*Fig V. Boxplots of Eigenvector values for PCA of phenotypic variables.* A) Boxplot of the eigenvector values with the colour fill indicating the measurement type (spore count, sporangia count, sporangia size), B) Boxplot of the eigenvector values with the colour fill indicating the temperature of the experiment for which the measurements were taken. Here, the central line of the boxplot indicates the median, the hinges indicate the 25^th^ and 75^th^ percentiles and the whiskers extending to the smallest/largest values no more than 1.5 * Interquartile range from the median.


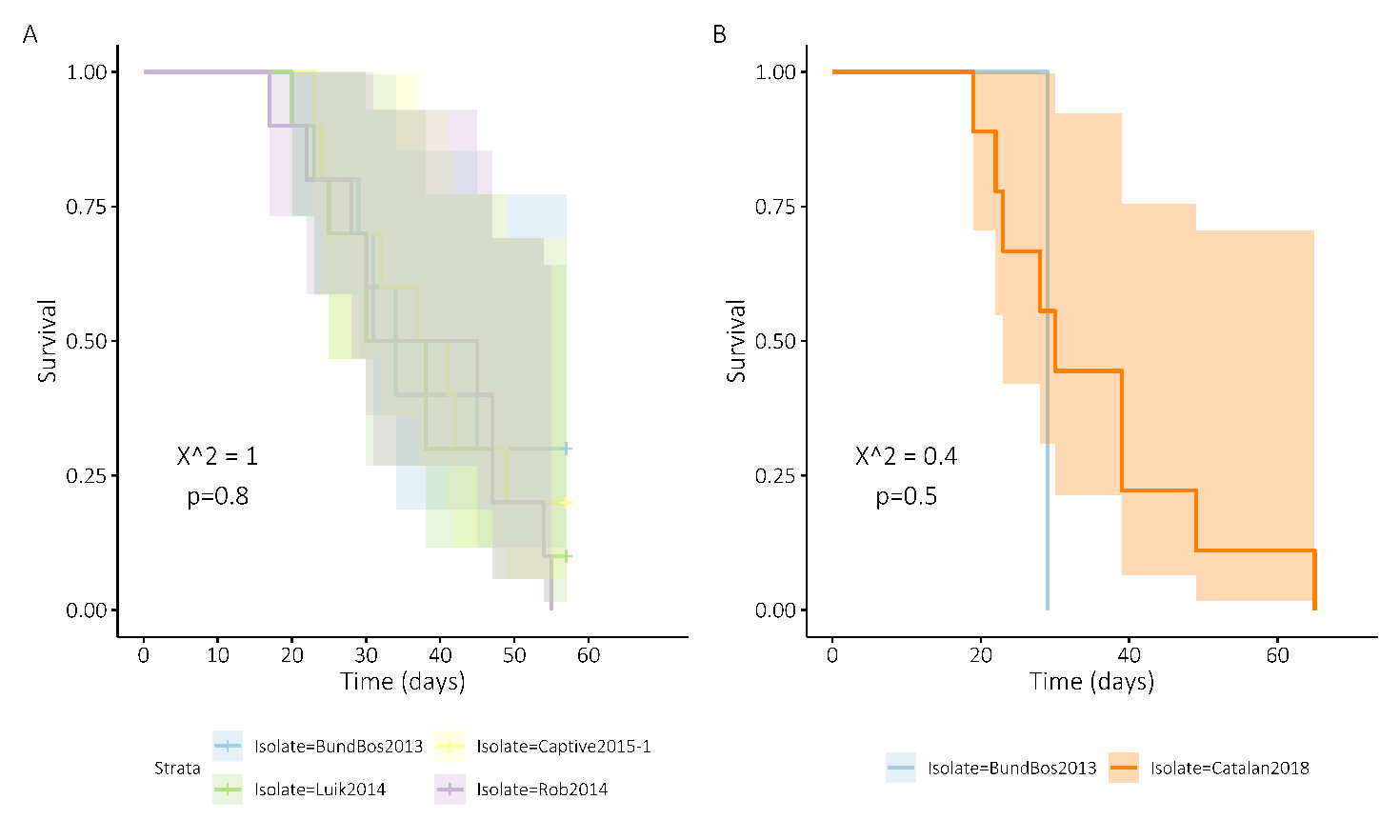


Fig W. Comparative survival analyses for Bsal isolates in Salamandra Salamandra (fire salamander). X^2^  and p-values calculated using the survdiff() and Surv() functions from the survival package in R. A) Stegen et al. (2017)[5] performed infection trials involving 4 Bsal isolates, B) Greener et al (2020)[6] performed infection trials using the Catalan2018 isolate with a BundBos2013 control. In both cases no statistically significant difference in mortality can be seen.


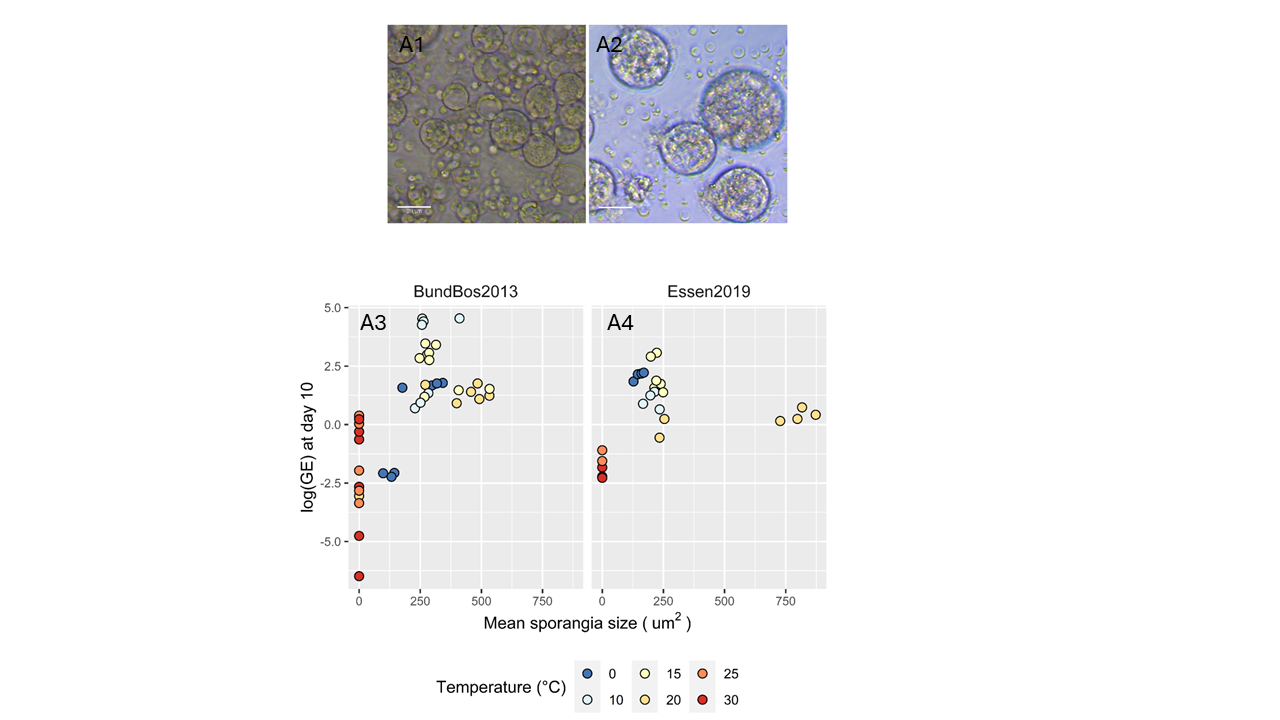


Fig X. Plasticity in sporangia size. B1) Photographic representation of BundBos2013 isolate at 20°C with “normal” sized sporangia, photographed at 20x magnification, scale bars represent 20µm. B2) Photographic representation of Essen2019 isolate at 20°C with “enlarged” sporangia, photographed at 20x magnification, scale bars represent 20µm. B3-B4) Bsal qPCR loads (log(genomic equivalents)) against mean sporangia size for wells containing BundBos2013 (B3) and Essen2019 (B4) at each temperature at day 10 (colour denotes incubation temperature), for Essen2019 wells with enlarged sporangia circled.


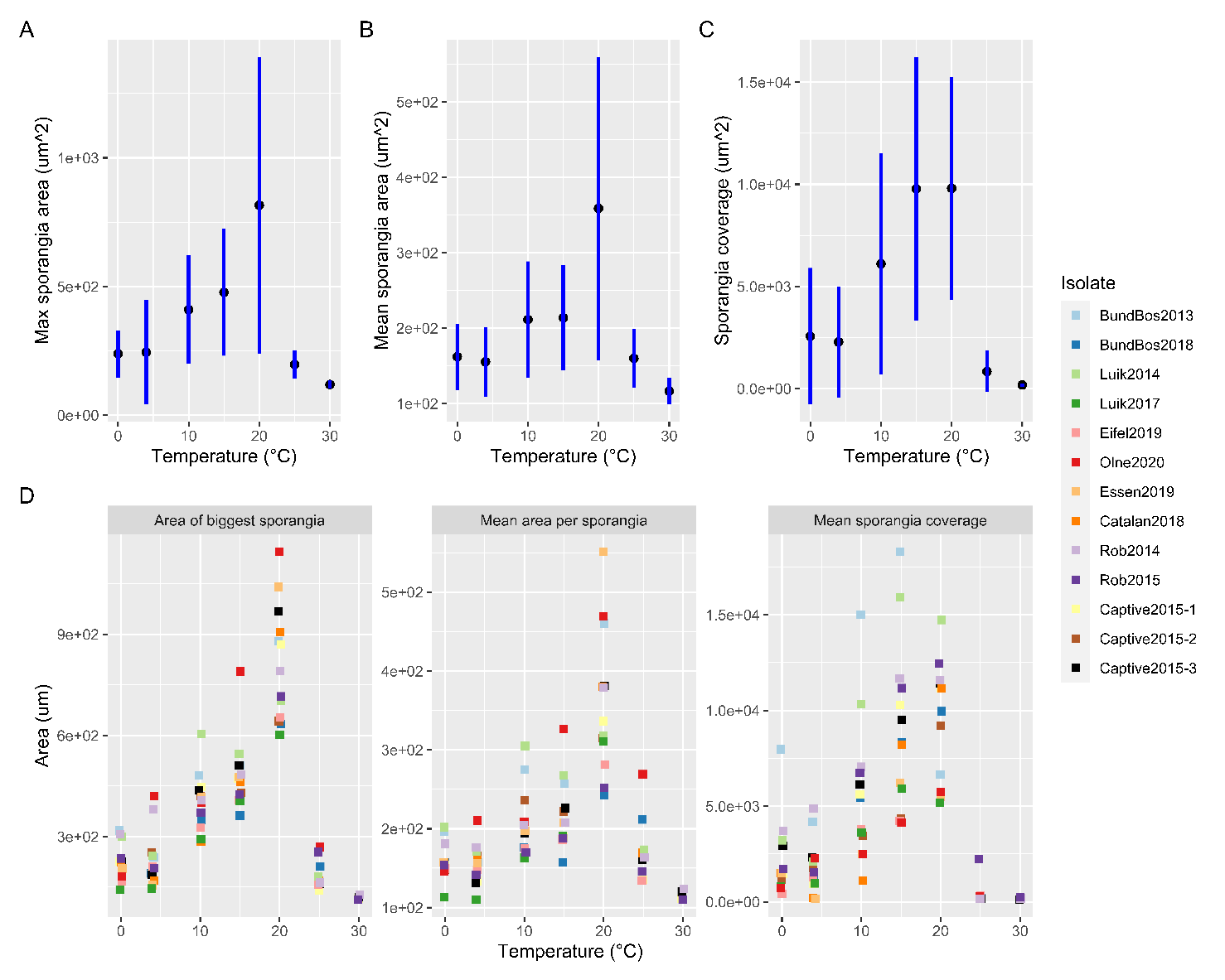


Fig Y. Sporangia size across a temperature gradient. Units for all area measurements in µm^2^ A-C) Mean (black dots) and 95% CI intervals (blue lines) calculated for all isolates combined across the tested temperature range for A) Maximum sporangia size – the largest sporangia observed in each image B) Mean sporangia size- the mean size of sporangia per image, C) Total sporangia coverage per image. D) Calculated per isolate across the temperature range, where colour denotes isolate identity, of the largest sporangia per image, the average of the mean size of sporangia per image and the mean sporangia coverage. Furthermore, 62 out of 65 of the five largest measurements of maximum sporangia size per isolate, and 59 of the five largest measurements of mean sporangia size per isolate, are at 20°C.

**Supplementary Tables**

**Table A. Details of Batrachochytrium salamandrivorans isolates included in analyses.** Locations of wild source populations are listed here and shown in Fig A; locations of captive isolates Captive2015-1, Captive2015-2 and Captive2015-3 are not included to maintain anonymity. All isolates presented in this dataset were collected from naturally infected fire salamanders (Salamandra salamandra) during their respective outbreak. Isolates were collected using protocols as described in Martel et al. (2013)[7].

| Isolate | Year of Isolation | Location | Passage | GenBank Sample | Accession Numbers |
| --- | --- | --- | --- | --- | --- |
| BundBos2013 | 2013 | Bunde, Netherlands | 14,20,26,29 | AMFP13/1 | PRJNA797022, GCA_021556675.1,  GCA_002006685.2 |
| Rob2014 | 2014 | Robertville, Belgium | 17 | AMFP14/1 | PRJNA610831 |
| Luik2014 | 2014 | Liege (Luik), Belgium | 10,22 | AMFP14/2 | PRJNA797021,  GCA_021556655.1 |
| Captive2015-1 | 2015 | Captive amphibian collection | 10,12 | AMFP15/1 | PRJNA610831, GCA_020617715.1 |
| Captive2015-2 | 2015 | Captive amphibian collection | 15 | AMFP15/2 | PRJNA610831, GCA_020617195.1 |
| Rob2015 | 2015 | Robertville, Belgium | 8,15 | AMFP15/3 | PRJNA610831,  GCA_020617725.1 |
| Captive2015-3 | 2015 | Captive amphibian collection | 6,9 | AMFP15/4 | PRJNA845763 |
| Luik2017 | 2017 | Liege (Luik), Belgium | 8,16 | AMFP17/1 | PRJNA610831 |
| BundBos2018 | 2018 | Bunde, Netherlands | 8,20 | AMFP18/1 | PRJNA610831 |
| Catalan2018 | 2018 | Montnegre i el Corredor Natural Park, Catalonia, Spain | 7,13 | AMFP18/2 | PRJNA610831, GCA_020617735.1 |
| Essen2019 | 2019 | Essen, Germany | 8,12 | AMFP19/1 | PRJNA845763,  JBGMNW000000000 |
| Eifel2019 | 2019 | Brandscheid (Watzbach), Eifel, Germany | 11 | AMFP19/2 | PRJNA845763 |
| Olne2020 | 2020 | Olne, Belgium | 4,7 | AMFP20/1 |  |

**Table B – *Bd* isolates included in genomic analysis**

| Sample | SRR | Lat. | Long. | Continent | Country | Species | Host family | Lineage | Year isolated |
| --- | --- | --- | --- | --- | --- | --- | --- | --- | --- |
| BLI1 | [SRR630154](https://trace.ncbi.nlm.nih.gov/Traces/sra/?run=SRR630154) | 47.459 | 7.783 | Europe | Switzerland | *Alytes obstetricans* | Alytidae | GPL | 2010 |
| TF5a1 | [SRR630604](https://trace.ncbi.nlm.nih.gov/Traces/sra/?run=SRR630604) | 39.858 | 2.837 | Europe | Spain | *Alytes muletensis* | Alytidae | CAPE | 2007 |
| AP15 | [SRR633648](https://trace.ncbi.nlm.nih.gov/Traces/sra/?run=SRR633648) | 40.058 | 9.203 | Europe | Italy | *Discoglossus sardus* | Alytidae | GPL | 2010 |
| 739 | SRR6375451 | 47.307 | 8.498 | Europe | Switzerland | *Alytes obstetricans* | Alytidae | CH | 2007 |
| BdBE6 | [SRR6375456](https://trace.ncbi.nlm.nih.gov/Traces/sra/?run=SRR6375456) | 50.76 | 5.768 | Europe | Belgium | *Ichthyosaura alpestris* | Salamandridae | GPL | 2016 |
| HR5 | [SRR6375545](https://trace.ncbi.nlm.nih.gov/Traces/sra/?run=SRR6375545) | 51.537 | -0.153 | Europe | UK | *Hyperolius riggenbachi* | Hyperoliidae | CAPE | 2013 |
| ADMALA | [SRR6378427](https://trace.ncbi.nlm.nih.gov/Traces/sra/?run=SRR6378427) | 36.873 | -4.062 | Europe | Spain | *Alytes dickhilleni* | Alytidae | GPL | 2012 |
| UKBER | [SRR6378438](https://trace.ncbi.nlm.nih.gov/Traces/sra/?run=SRR6378438) | 51.171 | -0.271 | Europe | UK | *Pelophylax ridibundus* | Ranidae | GPL | 2012 |
| CH4 | [SRR643908](https://trace.ncbi.nlm.nih.gov/Traces/sra/?run=SRR643908) | 47.013 | 7.793 | Europe | Switzerland | *Alytes obstetricans* | Alytidae | GPL | 2011 |

**Table C –** Coefficient estimates, confidence intervals and p-values of glms predicting GrowthCurver growth curve measurements with formulae glm(t_gen ~Isolate) and glm(r~Isolate), from GrowthCurver curves based on A) Zoosporangia counts, B) Total (zoospore and zoosporangia) counts

| **A – sporangia counts** | t_gen - Sporangia count doubling rate | | | r - growth rate | | |
| --- | --- | --- | --- | --- | --- | --- |
| Predictors | Estimates | CI | p | Estimates | CI | p |
| (Intercept) – BundBos2013 | 0.16 | -0.44 – 0.76 | 0.608 | 5.75 | 4.19 – 7.32 | **<0.001** |
| Isolate [BundBos2018] | 0.84 | -0.08 – 1.76 | 0.079 | -4.94 | -7.33 – -2.55 | **<0.001** |
| IsolateCaptive2015-1 | 0.59 | -0.33 – 1.51 | 0.217 | -4.17 | -6.56 – -1.79 | **0.001** |
| IsolateCaptive2015-2 | 0.78 | -0.26 – 1.83 | 0.147 | -1.75 | -4.46 – 0.95 | 0.209 |
| Isolate [Captive2015-3] | 0.7 | -0.22 – 1.62 | 0.139 | -2.67 | -5.06 – -0.28 | **0.033** |
| Isolate [Catalan2018] | 0.65 | -0.27 – 1.57 | 0.174 | -4.87 | -7.26 – -2.48 | **<0.001** |
| Isolate [Eifel2019] | 2.18 | 1.14 – 3.23 | **<0.001** | -5.42 | -8.12 – -2.71 | **<0.001** |
| Isolate [Essen2019] | 1.97 | 1.05 – 2.89 | **<0.001** | -5.36 | -7.74 – -2.97 | **<0.001** |
| Isolate [Luik2014] | 1.62 | 0.70 – 2.54 | **0.001** | -4.37 | -6.76 – -1.99 | **0.001** |
| Isolate [Luik2017] | 1.46 | 0.42 – 2.51 | **0.008** | -5.32 | -8.02 – -2.61 | **<0.001** |
| Isolate [Olne2020] | 0.15 | -0.89 – 1.20 | 0.775 | -0.5 | -3.21 – 2.20 | 0.718 |
| Isolate [Rob2014] | 1.23 | -0.12 – 2.57 | 0.079 | -5.14 | -8.63 – -1.65 | **0.006** |
| Isolate [Rob2015] | 0.24 | -0.68 – 1.16 | 0.617 | -3.71 | -6.10 – -1.33 | **0.004** |
| Observations | 68 |  |  | 68 |  |  |
| R2 Nagelkerke | 0.556 |  |  | 0.969 |  |  |

| **B – total counts** | t_gen - Sporangia count doubling rate | | | | r - growth rate | | |
| --- | --- | --- | --- | --- | --- | --- | --- |
| Predictors | Estimates | CI | p | Estimates | | CI | p |
| (Intercept) | 2.75 | 1.77 – 3.74 | **<0.001** | 0.57 | | -0.08 – 1.21 | 0.091 |
| Isolate [BundBos2018] | -1.81 | -3.21 – -0.42 | **0.013** | 0.24 | | -0.67 – 1.15 | 0.608 |
| Isolate[Captive2015-1] | -1.81 | -3.20 – -0.42 | **0.014** | 0.18 | | -0.73 – 1.09 | 0.701 |
| Isolate[Captive2015-2] | -1.59 | -3.15 – -0.03 | 0.05 | 0.07 | | -0.95 – 1.09 | 0.898 |
| Isolate[Captive2015-3] | -1.99 | -3.38 – -0.60 | **0.007** | 0.38 | | -0.54 – 1.29 | 0.421 |
| Isolate [Catalan2018] | -1.74 | -3.13 – -0.34 | **0.018** | 0.18 | | -0.73 – 1.09 | 0.703 |
| Isolate [Eifel2019] | 0.73 | -0.82 – 2.29 | 0.361 | -0.24 | | -1.27 – 0.78 | 0.641 |
| Isolate [Essen2019] | -1.69 | -3.08 – -0.30 | **0.021** | 0.19 | | -0.72 – 1.11 | 0.678 |
| Isolate [Luik2014] | -2 | -3.39 – -0.60 | **0.007** | 2.29 | | 1.37 – 3.20 | **<0.001** |
| Isolate [Luik2017] | 0.68 | -0.72 – 2.07 | 0.346 | -0.08 | | -0.99 – 0.83 | 0.862 |
| Isolate [Olne2020] | 2.79 | 1.24 – 4.35 | **0.001** | -0.43 | | -1.45 – 0.59 | 0.415 |
| Isolate [Rob2014] | -1.71 | -3.68 – 0.26 | 0.094 | 0.1 | | -1.19 – 1.39 | 0.881 |
| Isolate [Rob2015] | -1.59 | -2.98 – -0.20 | **0.029** | 0.1 | | -0.81 – 1.02 | 0.828 |
| Observations | 68 |  |  | 68 | |  |  |
| R2 Nagelkerke | 0.878 |  |  | 0.567 | |  |  |

**Table D –** output glm of fecundity at 15degrees (fecundity normalised by spore count at t=0 ~ Isolate * Time (treating day as a discrete variable)

|  | Estimate | Std. Error | t-value | Pr(>\|t\|) |  |  | Estimate | Std. Error | t-value | Pr(>\|t\|) |  |
| --- | --- | --- | --- | --- | --- | --- | --- | --- | --- | --- | --- |
| Int-Bundbos2013 | 1.02E-04 | 1.30E-04 | 0.78 | 0.44 |  | Rob2014:Day5 | 1.75E-04 | 3.47E-04 | 0.50 | 0.62 |  |
| BundBos2018 | 2.98E-04 | 1.94E-04 | 1.54 | 0.13 |  | Rob2015:Day5 | 1.19E-04 | 2.82E-04 | 0.42 | 0.67 |  |
| Captive2015-1 | 1.07E-04 | 1.94E-04 | 0.55 | 0.58 |  | BundBos2018:Day7 | 1.98E-04 | 2.82E-04 | 0.70 | 0.48 |  |
| Captive2015-2 | 5.39E-04 | 2.41E-04 | 2.23 | 0.03 | * | Captive2015-1:Day7 | 1.97E-04 | 2.82E-04 | 0.70 | 0.49 |  |
| Captive2015-3 | 1.38E-04 | 1.96E-04 | 0.71 | 0.48 |  | Captive2015-2:Day7 | 1.01E-03 | 3.47E-04 | 2.91 | 0.00 | ** |
| Catalan2018 | 1.88E-04 | 1.94E-04 | 0.97 | 0.33 |  | Captive2015-3:Day7 | 3.69E-04 | 2.85E-04 | 1.29 | 0.20 |  |
| Eifel2019 | 5.42E-04 | 2.41E-04 | 2.25 | 0.02 | * | Catalan2018:Day7 | 1.05E-04 | 2.82E-04 | 0.37 | 0.71 |  |
| Essen2019 | 1.82E-04 | 1.94E-04 | 0.94 | 0.35 |  | Eifel2019:Day7 | 7.60E-05 | 3.47E-04 | 0.22 | 0.83 |  |
| Luik2014 | -1.34E-05 | 1.99E-04 | -0.07 | 0.95 |  | Essen2019:Day7 | 4.41E-04 | 2.82E-04 | 1.56 | 0.12 |  |
| Luik2017 | 2.33E-03 | 2.11E-04 | 11.03 | 0.00 | *** | Luik2014:Day7 | 4.48E-04 | 2.85E-04 | 1.57 | 0.12 |  |
| Olne2020 | 3.23E-04 | 2.41E-04 | 1.34 | 0.18 |  | Luik2017:Day7 | -1.55E-03 | 3.05E-04 | -5.06 | 0.00 | *** |
| Rob2014 | 4.43E-05 | 2.41E-04 | 0.18 | 0.85 |  | Olne2020:Day7 | -2.35E-04 | 3.47E-04 | -0.68 | 0.50 |  |
| Rob2015 | 8.45E-05 | 1.94E-04 | 0.44 | 0.66 |  | Rob2014:Day7 | 6.92E-05 | 3.53E-04 | 0.20 | 0.84 |  |
| Day5 | -3.02E-05 | 1.96E-04 | -0.15 | 0.88 |  | Rob2015:Day7 | 1.26E-04 | 2.82E-04 | 0.45 | 0.66 |  |
| Day7 | 4.96E-05 | 1.96E-04 | 0.25 | 0.80 |  | BundBos2018:Day10 | -1.74E-04 | 2.79E-04 | -0.62 | 0.53 |  |
| Day10 | 3.52E-04 | 1.92E-04 | 1.84 | 0.07 | . | Captive2015-1: Day10 | -3.00E-04 | 2.79E-04 | -1.08 | 0.28 |  |
| BundBos2018:Day5 | 3.53E-04 | 2.82E-04 | 1.25 | 0.21 |  | Captive2015-2: Day10 | 7.52E-04 | 3.45E-04 | 2.18 | 0.03 | * |
| Captive2015-1: Day5 | 1.34E-04 | 2.82E-04 | 0.47 | 0.64 |  | Captive2015-3: Day10 | -1.83E-04 | 2.82E-04 | -0.65 | 0.52 |  |
| Captive2015-2: Day5 | 1.24E-03 | 3.47E-04 | 3.56 | 0.00 | *** | Catalan2018:Day10 | 6.40E-04 | 2.79E-04 | 2.29 | 0.02 | * |
| Captive2015-3: Day5 | 2.25E-04 | 2.84E-04 | 0.79 | 0.43 |  | Eifel2019:Day10 | -4.82E-04 | 3.45E-04 | -1.40 | 0.16 |  |
| Catalan2018:Day5 | 3.06E-04 | 2.82E-04 | 1.09 | 0.28 |  | Essen2019:Day10 | -2.42E-04 | 2.79E-04 | -0.87 | 0.39 |  |
| Eifel2019:Day5 | 2.92E-04 | 3.47E-04 | 0.84 | 0.40 |  | Luik2014:Day10 | 2.99E-05 | 2.82E-04 | 0.11 | 0.92 |  |
| Essen2019:Day5 | 3.80E-04 | 2.82E-04 | 1.35 | 0.18 |  | Luik2017:Day10 | -2.19E-03 | 3.03E-04 | -7.25 | 0.00 | *** |
| Luik2014:Day5 | 9.47E-04 | 2.85E-04 | 3.32 | 0.00 | *** | Olne2020:Day10 | -4.88E-04 | 3.45E-04 | -1.42 | 0.16 |  |
| Luik2017:Day5 | -1.44E-03 | 3.05E-04 | -4.72 | 0.00 | *** | Rob2014:Day10 | -3.55E-04 | 3.50E-04 | -1.01 | 0.31 |  |
| Olne2020:Day5 | -1.20E-04 | 3.47E-04 | -0.35 | 0.73 |  | Rob2015:Day10 | -2.38E-04 | 2.79E-04 | -0.85 | 0.39 |  |

**Table E** – Isolate response to temperatures- best fitting polynomials for *r* values extracted from GrowthCurver curves across temperatures

| BundBos2013 | y= -0.2282050250 + 0.4050027846x + -0.0311945087x**2+ 0.0006289786x**3 |
| --- | --- |
| BundBos2018 | y= 0.082746711 + 0.082241076x + -0.002422493x**2 |
| Captive2015-1 | y=1.005355e-01+ -8.651360e-02x + 2.504022e-02x**2+ -1.457744e-03x**3 + 2.402298e-05x**4 |
| Captive2015-2 | y= -0.07604377+ 0.12389778x + -0.00433720x**2 |
| Captive2015-3 | y=2.060481e-01+ -1.497330e-01x+ 4.652730e-02x**2+ -3.150899e-03x**3 + 6.106872e-05x**4 |
| Catalan2018 | y= 0.10499054+ 0.04791507x+ -0.00157575x**2 |
| Eifel2019 | y= -0.0123595855 + 0.3162210405x + -0.0447274496x**2+ 0.0024094428x**3 +-0.0000445818x**4 |
| Essen2019 | y=1.567552e-01+ -1.706901e-01x + 4.388142e-02x**2+ -2.836643e-03x**3 + 5.472548e-05x**4 |
| Luik2014 | y=-0.227376622+ 1.034672333x + -0.077913765x**2+ 0.001461052x**3 |
| Luik2017 | y=0.2970169063+ 0.7770911348x+ -0.1397071745x**2+ 0.0080093548x**3 +-0.0001405729x**4 |
| Olne2020 | y=0.2832663089+ -0.0792349185x+ 0.0075629206x**2+ -0.0001643377x**3 |
| Rob2014 | y= 0.118192191+ 0.067270239x+ -0.002407837x**2 |
| Rob2015 | y=1.452617e-01+ -4.746493e-02x+ 1.781501e-02x**2 + -1.133214e-03x**3 + 1.984147e-05x**4 |

**Table F**. Estimates, confidence intervals and p value output from a beta regression model with formula proportion of immotile spores ~ Isolate * *r* value

|  | Surface spore proportion | | |
| --- | --- | --- | --- |
| Predictors | Estimates | CI | *P* |
| **(Intercept)** | **0.04** | **0.03–0.05** | **<0.001** |
| r | 1.06 | 0.96–1.16 | 0.236 |
| Isolate[BundBos2018] | 0.8 | 0.54–1.18 | 0.265 |
| **Isolate[Captive2015-1]** | **1.57** | **1.12–2.20** | **0.009** |
| Isolate[Captive2015-2] | 1.05 | 0.72–1.55 | 0.785 |
| **Isolate[Captive2015-3]** | **1.42** | **1.08–1.87** | **0.013** |
| **Isolate[Catalan2018]** | **2.36** | **1.75–3.18** | **<0.001** |
| Isolate[Eifel2019] | 1.3 | 0.95–1.78 | 0.097 |
| **Isolate[Essen2019]** | **1.36** | **1.04–1.78** | **0.025** |
| Isolate[Luik2014] | 0.89 | 0.66–1.18 | 0.407 |
| **Isolate[Luik2017]** | **2.78** | **2.25–3.42** | **<0.001** |
| **Isolate[Olne2020]** | **2.24** | **1.73–2.89** | **<0.001** |
| **Isolate[Rob2014]** | **1.45** | **1.07–1.96** | **0.017** |
| **Isolate[Rob2015]** | **2.28** | **1.57–3.29** | **<0.001** |
| **r:Isolate[BundBos2018]** | **2.87** | **1.63–5.05** | **<0.001** |
| **r:Isolate[Captive2015-1]** | **2.6** | **1.43–4.75** | **0.002** |
| **r:Isolate[Captive2015-2]** | **0.31** | **0.17–0.54** | **<0.001** |
| r:Isolate[Captive2015-3] | 1.26 | 0.97–1.63 | 0.088 |
| r:Isolate[Catalan2018] | 0.57 | 0.33–1.00 | 0.051 |
| **r:Isolate[Eifel2019]** | **0.36** | **0.23–0.57** | **<0.001** |
| r:Isolate[Essen2019] | 1.11 | 0.84–1.48 | 0.458 |
| r:Isolate[Luik2014] | 1.13 | 1.00–1.29 | 0.05 |
| **r:Isolate[Luik2017]** | **0.87** | **0.77–0.99** | **0.032** |
| **r:Isolate[Olne2020]** | **0.53** | **0.30–0.91** | **0.021** |
| r:Isolate[Rob2014] | 1 | 0.77–1.28 | 0.969 |
| **r:Isolate[Rob2015]** | **2.59** | **1.25–5.37** | **0.01** |
| Observations | 1513 |  |  |
| R2 | 0.202 |  |  |

**Table G** – posthoc pairwise comparisons of isolate spore attachment from model described in Fig B, estimates give on log scale, multiple test correction of p-values applied using Tukeys method. Est. = estimate, SE = standard error.

| Pairwise comparison | est. | SE | t.ratio | p.value | Pairwise comparison | est. | SE | t.ratio | p.value |
| --- | --- | --- | --- | --- | --- | --- | --- | --- | --- |
| BundBos2013 - BundBos2018 | 0.33 | 0.17 | 1.96 | 0.7590 | (Captive2015-2) - Olne2020 | -0.42 | 0.25 | -1.65 | 0.9121 |
| BundBos2013 - (Captive2015-1) | -0.12 | 0.17 | -0.69 | 1.0000 | (Captive2015-2) - Rob2014 | -0.57 | 0.27 | -2.12 | 0.6536 |
| **BundBos2013 - (Captive2015-2)** | **0.75** | **0.22** | **3.42** | **0.0397** | **(Captive2015-2) - Rob2015** | **-1.22** | **0.23** | **-5.24** | **<.0001** |
| BundBos2013 - (Captive2015-3) | 0.32 | 0.17 | 1.85 | 0.8234 | (Captive2015-3) - Catalan2018 | -0.33 | 0.19 | -1.73 | 0.8789 |
| BundBos2013 - Catalan2018 | -0.01 | 0.17 | -0.07 | 1.0000 | **(Captive2015-3) - Eifel2019** | **0.90** | **0.24** | **3.82** | **0.0105** |
| **BundBos2013 - Eifel2019** | **1.22** | **0.22** | **5.50** | **<.0001** | (Captive2015-3) - Essen2019 | 0.36 | 0.19 | 1.91 | 0.7907 |
| **BundBos2013 - Essen2019** | **0.68** | **0.17** | **3.95** | **0.0064** | **(Captive2015-3) - Luik2014** | **-0.66** | **0.19** | **-3.50** | **0.0304** |
| BundBos2013 - Luik2014 | -0.34 | 0.17 | -2.03 | 0.7110 | (Captive2015-3) - Luik2017 | 0.46 | 0.19 | 2.41 | 0.4433 |
| **BundBos2013 - Luik2017** | **0.77** | **0.17** | **4.50** | **0.0007** | (Captive2015-3) - Olne2020 | 0.02 | 0.21 | 0.08 | 1.0000 |
| BundBos2013 - Olne2020 | 0.33 | 0.20 | 1.70 | 0.8939 | (Captive2015-3) - Rob2014 | -0.14 | 0.23 | -0.59 | 1.0000 |
| BundBos2013 - Rob2014 | 0.18 | 0.22 | 0.83 | 0.9998 | **(Captive2015-3) - Rob2015** | **-0.79** | **0.19** | **-4.18** | **0.0027** |
| BundBos2013 - Rob2015 | -0.47 | 0.17 | -2.78 | 0.2180 | **Catalan2018 - Eifel2019** | **1.23** | **0.24** | **5.21** | **<.0001** |
| BundBos2018 - (Captive2015-1) | -0.45 | 0.19 | -2.39 | 0.4517 | **Catalan2018 - Essen2019** | **0.69** | **0.19** | **3.64** | **0.0195** |
| BundBos2018 - (Captive2015-2) | 0.41 | 0.23 | 1.77 | 0.8632 | Catalan2018 - Luik2014 | -0.33 | 0.19 | -1.77 | 0.8627 |
| BundBos2018 - (Captive2015-3) | -0.02 | 0.19 | -0.10 | 1.0000 | **Catalan2018 - Luik2017** | **0.79** | **0.19** | **4.14** | **0.0032** |
| BundBos2018 - Catalan2018 | -0.35 | 0.19 | -1.83 | 0.8309 | Catalan2018 - Olne2020 | 0.34 | 0.21 | 1.63 | 0.9202 |
| **BundBos2018 - Eifel2019** | **0.88** | **0.24** | **3.74** | **0.0139** | Catalan2018 - Rob2014 | 0.19 | 0.23 | 0.83 | 0.9998 |
| BundBos2018 - Essen2019 | 0.34 | 0.19 | 1.81 | 0.8451 | Catalan2018 - Rob2015 | -0.46 | 0.19 | -2.45 | 0.4159 |
| **BundBos2018 - Luik2014** | **-0.68** | **0.19** | **-3.60** | **0.0220** | Eifel2019 - Essen2019 | -0.54 | 0.24 | -2.28 | 0.5364 |
| BundBos2018 - Luik2017 | 0.44 | 0.19 | 2.31 | 0.5146 | **Eifel2019 - Luik2014** | **-1.56** | **0.24** | **-6.63** | **<.0001** |
| BundBos2018 - Olne2020 | 0.00 | 0.21 | -0.01 | 1.0000 | Eifel2019 - Luik2017 | -0.44 | 0.24 | -1.87 | 0.8109 |
| BundBos2018 - Rob2014 | -0.16 | 0.23 | -0.67 | 1.0000 | **Eifel2019 - Olne2020** | **-0.88** | **0.25** | **-3.48** | **0.0329** |
| **BundBos2018 - Rob2015** | **-0.81** | **0.19** | **-4.28** | **0.0018** | **Eifel2019 - Rob2014** | **-1.04** | **0.27** | **-3.83** | **0.0101** |
| **(Captive2015-1) - (Captive2015-2)** | **0.86** | **0.23** | **3.71** | **0.0151** | **Eifel2019 - Rob2015** | **-1.69** | **0.24** | **-7.17** | **<.0001** |
| (Captive2015-1) - (Captive2015-3) | 0.43 | 0.19 | 2.29 | 0.5233 | **Essen2019 - Luik2014** | **-1.02** | **0.19** | **-5.40** | **<.0001** |
| (Captive2015-1) - Catalan2018 | 0.11 | 0.19 | 0.56 | 1.0000 | Essen2019 - Luik2017 | 0.10 | 0.19 | 0.50 | 1.0000 |
| **(Captive2015-1) - Eifel2019** | **1.33** | **0.24** | **5.66** | **<.0001** | Essen2019 - Olne2020 | -0.35 | 0.21 | -1.63 | 0.9191 |
| **(Captive2015-1) - Essen2019** | **0.80** | **0.19** | **4.20** | **0.0025** | Essen2019 - Rob2014 | -0.50 | 0.23 | -2.15 | 0.6291 |
| (Captive2015-1) - Luik2014 | -0.23 | 0.19 | -1.21 | 0.9922 | **Essen2019 - Rob2015** | **-1.15** | **0.19** | **-6.08** | **<.0001** |
| **(Captive2015-1) - Luik2017** | **0.89** | **0.19** | **4.70** | **0.0003** | **Luik2014 - Luik2017** | **1.12** | **0.19** | **5.90** | **<.0001** |
| (Captive2015-1) - Olne2020 | 0.45 | 0.21 | 2.13 | 0.6450 | Luik2014 - Olne2020 | 0.68 | 0.21 | 3.21 | 0.0745 |
| (Captive2015-1) - Rob2014 | 0.30 | 0.23 | 1.28 | 0.9871 | Luik2014 - Rob2014 | 0.52 | 0.23 | 2.27 | 0.5418 |
| (Captive2015-1) - Rob2015 | -0.35 | 0.19 | -1.89 | 0.8028 | Luik2014 - Rob2015 | -0.13 | 0.19 | -0.68 | 1.0000 |
| (Captive2015-2) - (Captive2015-3) | -0.43 | 0.23 | -1.85 | 0.8222 | Luik2017 - Olne2020 | -0.44 | 0.21 | -2.08 | 0.6796 |
| (Captive2015-2) - Catalan2018 | -0.76 | 0.23 | -3.26 | 0.0642 | Luik2017 - Rob2014 | -0.59 | 0.23 | -2.56 | 0.3411 |
| (Captive2015-2) - Eifel2019 | 0.47 | 0.27 | 1.72 | 0.8848 | **Luik2017 - Rob2015** | **-1.24** | **0.19** | **-6.57** | **<.0001** |
| (Captive2015-2) - Essen2019 | -0.07 | 0.23 | -0.30 | 1.0000 | Olne2020 - Rob2014 | -0.15 | 0.25 | -0.61 | 1.0000 |
| **(Captive2015-2) - Luik2014** | **-1.09** | **0.23** | **-4.69** | **0.0003** | **Olne2020 - Rob2015** | **-0.80** | **0.21** | **-3.81** | **0.0108** |
| (Captive2015-2) - Luik2017 | 0.03 | 0.23 | 0.11 | 1.0000 | Rob2014 - Rob2015 | -0.65 | 0.23 | -2.82 | 0.2000 |

**Table H** – Summary of structural variants for 5 isolates, identified by Assemblytics, based on assembly alignment to the reference BundBos2013 assembly (GCA_002006685.2), annotated by size of element. Sequences were aligned as per Assemblytics requirements (see methods).

|  |  | **Luik2014** | **Captive2015-1** | **Captive2015-2** | **Rob2015** | **Catalan2018** |
| --- | --- | --- | --- | --- | --- | --- |
|  | **Size range (bp)** | **Total bp** | **Total bp** | **Total bp** | **Total bp** | **Total bp** |
|  | Genome size | 4.139E+07 | 3.559E+07 | 3.492E+07 | 3.836E+07 | 4.004E+07 |
|  | Aligned | 2.764E+07 | 3.414E+07 | 3.300E+07 | 3.614E+07 | 3.867E+07 |
| Insertion | 50-500 | 1658 | 2695 | 3123 | 2483 | 2483 |
|  | 500-10000 | 23969 | 8870 | 4795 | 19595 | 19595 |
| Deletion | 50-500 | 1536 | 1579 | 2649 | 2518 | 2518 |
|  | 500-10000 | 23414 | 39929 | 49857 | 19442 | 19442 |
| Tandem expansion | 50-500 | 1173 | 377 | 1680 | 948 | 948 |
|  | 500-10000 | 14171 | 4074 | 23621 | 23883 | 23883 |
| Tandem contraction | 50-500 | 0 | 553 | 123 | 0 | 0 |
|  | 500-10000 | 19749 | 35001 | 37762 | 25024 | 25024 |
| Repeat expansion | 50-500 | 3147 | 2155 | 2840 | 3293 | 3293 |
|  | 500-10000 | 69385 | 13257 | 12902 | 30207 | 30207 |
| Repeat contraction | 50-500 | 2996 | 1154 | 1237 | 1376 | 1376 |
|  | 500-10000 | 51715 | 50840 | 67339 | 106024 | 106024 |
|  | Total for all variants | 212913 | 160484 | 207928 | 234793 | 234793 |

References

1. Kelly M, Pasmans F, Muñoz JF, Shea TP, Carranza S, Cuomo CA, et al. Diversity, multifaceted evolution, and facultative saprotrophism in the European Batrachochytrium salamandrivorans epidemic. Nat Commun. 2021;12: 6688. doi:10.1038/s41467-021-27005-0

2. Gastebois A, Aimanianda V, Bachellier-Bassi S, Nesseir A, Firon A, Beauvais A, et al. SUN proteins belong to a novel family of β-(1,3)-glucan-modifying enzymes involved in fungal morphogenesis. J Biol Chem. 2013;288: 13387–13396. doi:10.1074/jbc.M112.440172

3. Li Y, Heller J, Gonçalves AP, Glass NL. The Predicted Mannosyltransferase GT69-2 Antagonizes RFW-1 To Regulate Cell Fusion in Neurospora crassa. mBio. 12: e00307-21. doi:10.1128/mBio.00307-21

4. Muszewska A, Stepniewska-Dziubinska MM, Steczkiewicz K, Pawlowska J, Dziedzic A, Ginalski K. Fungal lifestyle reflected in serine protease repertoire. Sci Rep. 2017;7: 9147. doi:10.1038/s41598-017-09644-w

5. Stegen G, Pasmans F, Schmidt BR, Rouffaer LO, Van Praet S, Schaub M, et al. Drivers of salamander extirpation mediated by Batrachochytrium salamandrivorans. Nature. 2017;544: 353–356. doi:10.1038/nature22059

6. Greener MS, Verbrugghe E, Kelly M, Blooi M, Beukema W, Canessa S, et al. Presence of low virulence chytrid fungi could protect European amphibians from more deadly strains. Nature communications. 2020;11: 1–11.

7. Martel A, Sluijs AS der, Blooi M, Bert W, Ducatelle R, Fisher MC, et al. Batrachochytrium salamandrivorans sp. nov. causes lethal chytridiomycosis in amphibians. PNAS. 2013;110: 15325–15329. doi:10.1073/pnas.1307356110
